# Comparison of Intact *Arabidopsis thaliana* Leaf Transcript Profiles during Treatment with Inhibitors of Mitochondrial Electron Transport and TCA Cycle

**DOI:** 10.1371/journal.pone.0044339

**Published:** 2012-09-18

**Authors:** Ann L. Umbach, Jelena Zarkovic, Jianping Yu, Michael E. Ruckle, Lee McIntosh, Jeffery J. Hock, Scott Bingham, Samuel J. White, Rajani M. George, Chalivendra C. Subbaiah, David M. Rhoads

**Affiliations:** 1 Department of Biology, Duke University, Durham, North Carolina, United States of America; 2 School of Life Sciences, Arizona State University, Tempe, Arizona, United States of America; 3 Department of Energy Plant Research Laboratory and the Department of Biochemistry and Molecular Biology, Michigan State University, East Lansing, Michigan, United States of America; 4 School of Plant Sciences, University of Arizona, Tucson, Arizona, United States of America; Nanjing Agricultural University, China

## Abstract

Plant mitochondria signal to the nucleus leading to altered transcription of nuclear genes by a process called mitochondrial retrograde regulation (MRR). MRR is implicated in metabolic homeostasis and responses to stress conditions. Mitochondrial reactive oxygen species (mtROS) are a MRR signaling component, but whether all MRR requires ROS is not established. Inhibition of the cytochrome respiratory pathway by antimycin A (AA) or the TCA cycle by monofluoroacetate (MFA), each of which initiates MRR, can increase ROS production in some plant cells. We found that for AA and MFA applied to leaves of soil-grown *Arabidopsis thaliana* plants, ROS production increased with AA, but not with MFA, allowing comparison of transcript profiles under different ROS conditions during MRR. Variation in transcript accumulation over time for eight nuclear encoded mitochondrial protein genes suggested operation of both common and distinct signaling pathways between the two treatments. Consequences of mitochondrial perturbations for the whole transcriptome were examined by microarray analyses. Expression of 1316 and 606 genes was altered by AA and MFA, respectively. A subset of genes was similarly affected by both treatments, including genes encoding photosynthesis-related proteins. MFA treatment resulted in more down-regulation. Functional gene category (MapMan) and cluster analyses showed that genes with expression levels affected by perturbation from AA or MFA inhibition were most similarly affected by biotic stresses such as pathogens. Overall, the data provide further evidence for the presence of mtROS-independent MRR signaling, and support the proposed involvement of MRR and mitochondrial function in plant responses to biotic stress.

## Introduction

Plant mitochondria and chloroplasts communicate with the cell nucleus to modify nuclear gene expression so that organelle and cell properties can be adjusted as metabolism and the environment change. For mitochondria, this signaling is termed mitochondrial retrograde regulation (MRR) [1], [2]. Reactive oxygen species (ROS) are generated by mitochondria (mtROS) as part of normal metabolism [3]–[6] and mtROS appear to be signaling intermediates in MRR when mitochondrial function is perturbed [4], [7], [8]. MRR could be involved in plant response to stress because increases in mtROS have been associated with various biotic and abiotic stresses in plants [4], [9]. In addition to mtROS, mitochondrial calcium has been identified as a likely MRR signaling component [10]. Whether mtROS, calcium and/or other molecules are necessary for all MRR, which nuclear genes are affected by MRR, and how much MRR contributes to the response of plants to environmental stresses are subjects of ongoing study.

Plants with mutations in genes encoding mitochondrial electron transport chain (mtETC) components demonstrate the importance of mitochondria for many processes. Different Complex I mutations alone affect chloroplasts [11], cold acclimation [12], and development and stress resistance [13]–[15]. Large scale disruption of the mitochondrial genome can also make plants more heat tolerant [16]. However, for these and most other stable mutations causing mitochondrial dysfunction, whether an observed effect results directly from altered MRR or indirectly from compensatory mechanisms or metabolic limitations is difficult to discern because the mutant plants are in a steady state [17].

In one approach to the analysis of MRR, chemicals applied to leaves or suspension culture cells have been assessed for their ability to alter transcription of nuclear genes. Most work has focused on nuclear genes encoding mitochondrial proteins (NEMP genes), particularly genes for alternative NAD(P)H dehydrogenases (NDHs) and for alternative oxidases (AOXs). Together, NDH and AOX make a non-phosphorylating bypass pathway for the cytochrome pathway of the mtETC [18], and, accordingly, specific genes for AOXs and NDHs are often induced coordinately [19]–[22].

Two exogenous chemical treatments that may mimic MRR signals are H_2_O_2_, representing increased mtROS production, and organic acids that are part of the TCA cycle, including citrate and malate. These treatments all induce AOX [8], [19], [22]–[25] and NDH genes [19], [22]. The organic acids can induce AOX genes without a marked increase in cellular ROS (tobacco, *Nicotiana tabacum* [24]; soybean, *Glycine max* [23]), indicating that ROS-independent pathways inducing *AOX* and *NDH*, as well as ROS-dependent ones, operate in cells. These results with organic acids have been interpreted more specifically as indicating that *mt*ROS-independent MRR pathways also exist. However, it is not clear that the measured responses to either of these exogenous chemical stimuli accurately represent mitochondrial signaling, whether mtROS-dependent or -independent. AOX gene expression was thought to be responsive only to mitochondrial signals, but *AOX1a* of Arabidopsis is now known to respond to non-mitochondrial as well as mitochondrial signaling pathways and so is not an obligate MRR marker [26]. Indirect evidence suggests this is the case for the NDH and other NEMP genes also [19], [22]. Further, H_2_O_2_ acts as a signal for various subcellular sites in addition to mitochondria [27]. Similarly, organic acids occur in various cellular compartments for which they may be signaling molecules, and their effects on AOX genes could be due to changes in general carbon availability rather than specific signaling [24]. These considerations make results with either H_2_O_2_ or organic acids difficult to interpret and leave the existence of mtROS-independent MRR pathways unresolved.

A related experimental approach better ensures that mitochondrial perturbation, with consequent initiation of MRR, is the primary starting point for changes in nuclear gene transcription. This approach uses the application of known mitochondrial inhibitors. Inhibitors of all the mtETC complexes, including antimycin A (AA) which inhibits Complex III, and MFA (monofluoroacetate), a TCA cycle inhibitor that acts on aconitase, induce expression of genes encoding AOX (e.g., [19], [21]–[23], [25], [28]–[31]) and many also induce genes encoding NDHs [19], [22]. *AtAOX1a* transcript accumulation kinetics vary [29], and distinct AOX genes are induced [32], depending on which mtETC complex is inhibited. These and other studies [8], [21], [24], [33], [34] indicate that MRR can arise from a variety of mitochondrial perturbations, through different signaling pathways. Whether or not any of these pathways operate independently of mtROS specifically or cellular ROS in general is not addressed by these studies. Typically ROS are not measured during inhibitor treatments; when they have been measured, only increases have been found (AA treatment of suspension culture cells; Arabidopsis [35], [36]; soybean [23]; tobacco [7], [8], [24]; MFA treatment of tobacco suspension culture cells [8], [24]). Inhibitor-induced mitochondrial perturbation without increased ROS production, and its consequences for mitochondrial signaling and MRR, has yet to be examined.

Beyond *AOX*, *NDH*, and other NEMP genes [19], [22], [30], [37], few studies have addressed the possible scope of MRR and impaired mitochondrial function on nuclear gene expression following mitochondrial inhibition. With rotenone, a Complex I inhibitor, comprehensive transcript analyses have been done in Arabidopsis [17], [21], but with malonate, a Complex II (succinate dehydrogenase) inhibitor, one reporter gene was followed [4] and for AA treatment, only small numbers of non-NEMP genes have been monitored in tobacco suspension culture cells [8], [38] and in excised Arabidopsis leaves [22]. One study using AA examined a large, but partial, gene set in excised Arabidopsis leaves [39], but AA effects were not distinguishable from those of leaf wounding or submergence. For the alga *Chlamydomonas reinhardtii*, the effects AA on the transcriptome were examined in conjunction with application of acetate [40].

To address further the relationship of MRR and nuclear gene expression, we sought to survey and compare potential MRR targets during mtETC inhibition and during TCA cycle inhibition. For this we used intact Arabidopsis plant leaves treated with AA or with MFA and found these treatments had distinct consequences for ROS production. AA did increase ROS production, as has been demonstrated in other systems. However, unlike for previous observations (see above), MFA treatment did not detectably increase ROS production. This allowed us to compare kinetics of transcript accumulation of selected NEMP genes under circumstances of unchanged and elevated ROS during known mitochondrial disruptions, providing evidence for MRR without dramatic elevation of ROS. We also compared the whole transcriptomes of treated leaves to examine the scope of the response of nuclear genes to the restriction of mitochondrial function by these two inhibitors. Analyses indicate many gene targets are affected, either directly or indirectly, by MRR from reduced mitochondrial function. For either treatment, regardless of ROS level, a strong response to oxidative stress by the transcriptome was not detected.

## Results

### Tissue ROS Measurements

**Figure 1:**
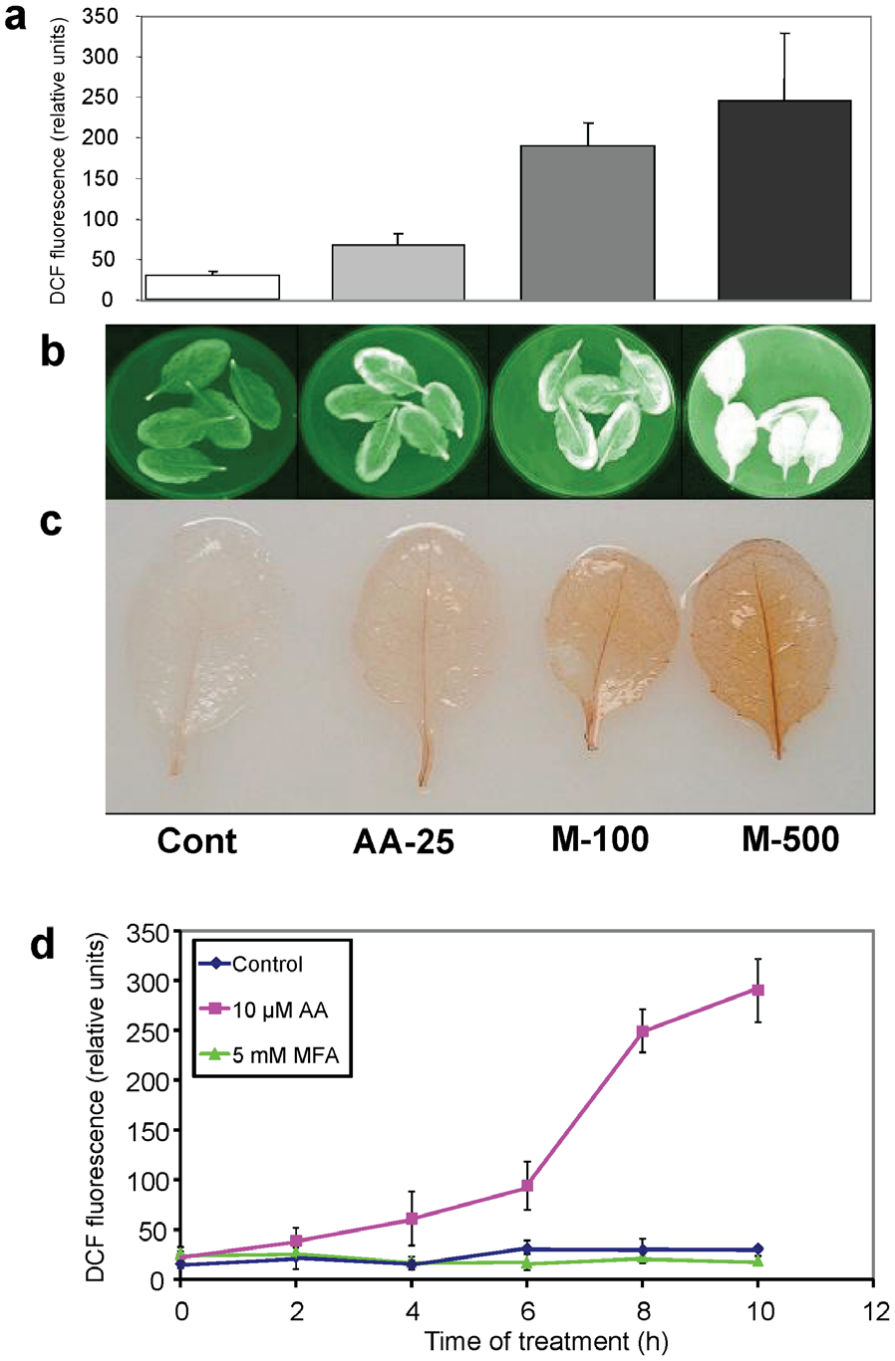
DCF fluorescence used to measure ROS levels from excised leaves. Leaves were treated as detailed in ‘Materials and Methods.’ Samples from left to right in panels (a) to (c): control leaves (Cont), leaves treated for 6 h with 25 µM AA (AA-25), with 100 µM menadione (M-100), or with 500 µM menadione (M-500). a, DCF fluorescence in the incubation medium, measured using a plate reader. Averages and standard deviations from three independent tests are shown. b, Imaging of *in vivo* DCF fluorescence using a Kodak image station. c, DAB staining of treated leaves. d, DCF fluorescence in the incubation medium was used to measure ROS production in leaves that were either incubated in medium with H_2_DCFDA alone (diamonds) or in medium with H_2_DCFDA plus 10 µM AA (squares) or 5 mM MFA (triangles) in the dark. The graphed points are averages of three separate bioreplicate experiments. For each experiment, at each time point, three aliquots were removed from the incubation medium of each of three separate samples and fluorescence was measured as described in ‘Materials and Methods.’ Error bars show the standard error of the mean of the three bioreplicate experiments and, where bars are not visible, do not exceed the symbol size.

Measurement of oxidized 2′,7′-dichlorofluorescein (DCF) in the external medium has been used with plant cells to assess cellular ROS production, specifically H_2_O_2_ and other peroxides [7], [24], [41], [42]. We adapted this technique to leaves under two inhibitor treatment conditions, one using intact leaves and one using excised leaves, with the same qualitative results. Menadione (vitamin K), a pro-oxidant that generates ROS upon reaction with cellular components [36], was used as a positive control with excised leaves. DCF fluorescence in the medium from excised leaves incubated with reduced DCF-diacetate (H_2_DCFDA) alone or H_2_DCFDA plus AA or menadione for 6 h in the dark correlated well with imaging of leaves for DCF fluorescence and with diaminobenzidine (DAB) leaf staining, which also detects H_2_O_2_ (Fig. 1a–c). These results indicated that fluorescence of DCF equilibrated with the medium is an effective and convenient measure of tissue ROS production.

The technique was used to determine ROS level changes in excised leaves during AA or MFA inhibition for up to 10 h in the dark. ROS production increased in leaves incubated in 10 µM AA by 4 h, continuing to 10 h (Fig. 1d). In contrast, ROS levels in control samples and samples incubated in 5 mM MFA were similar throughout the experiment (Fig. 1d).

**Figure 2:**
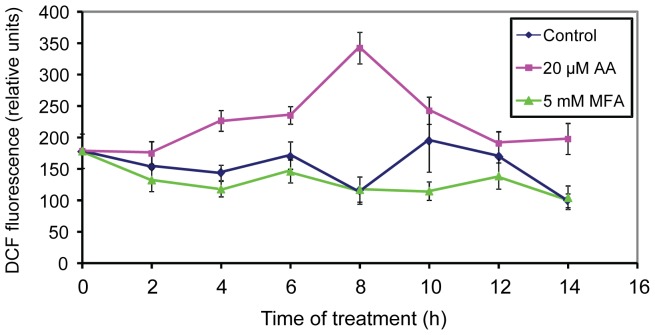
DCF fluorescence used to measure ROS production over time in leaves of intact plants treated with 5 mM MFA (triangles) or 20 µM AA (squares) or control treated (diamonds). At each time point after inhibitor application, leaves were harvested and incubated with H_2_DCFDA. DCF fluorescence was measured in aliquots from the incubation medium as described in ‘Materials and Methods.’ The graphed points are averages of three separate bioreplicate experiments. In each experiment, measurements were made for each of three replicates from plants in independent pots. Error bars show the standard error among the three experiments.

For gene transcript experiments (see below), intact plants were exposed to inhibitors by sprayed application, which initiated the exposure period (see ‘Materials and Methods’). When ROS production by leaves treated on intact plants was measured, AA treatment (20 µM) resulted in increased ROS production, which peaked at 8 h, while with 5 mM MFA, measured ROS levels were at or below those of the controls through 14 h of measurements (Fig. 2).

### NEMP Gene Transcript Accumulation Time Courses with AA or MFA Treatment

The transcript accumulation kinetics of several NEMP genes were followed over a 12 h time course. These NEMP genes encode: two mitochondrial innermembrane proteins that help to bypass the mtETC [*AtAOX1a* (At3g22370), encoding the most highly expressed AOX isozyme, and *NDB2* (At4g05020), encoding an NDH localized to the external surface of the inner mitochondrial membrane], two subunits of succinate dehydrogenase, an enzyme involved in both the mtETC and the TCA cycle [*SDH-FP* (At5g66760), the flavoprotein, and *SDH2-1* (At3g27380), the iron sulfur subunit], three proteins associated with stress [*GDH2* (At5g07440), glutamate dehydrogenase 2; *mtGST* (At1g02930), a glutathione *S*-transferase that is associated with Arabidopsis mitochondria [43]; and *HSP70-9* (At4g37910), a mitochondrial heat shock protein], and *mtPORIN* (At5g15090), which is part of the permeability transition pore that may control programmed cell death through MRR in response to stresses [2], [8]. In addition to previous studies (e.g. [39]) and/or our preliminary microarray results suggesting that each responds to MRR, these genes were chosen due to their potential importance in helping mitochondria respond to mitochondrial dysfunctions associated with stresses. For convenience throughout, we use “induction” synonymously with “up-regulation,” meaning increased transcript accumulation; and “repression” synonymously with “down-regulation,” meaning decreased transcript accumulation.

**Figure 3:**
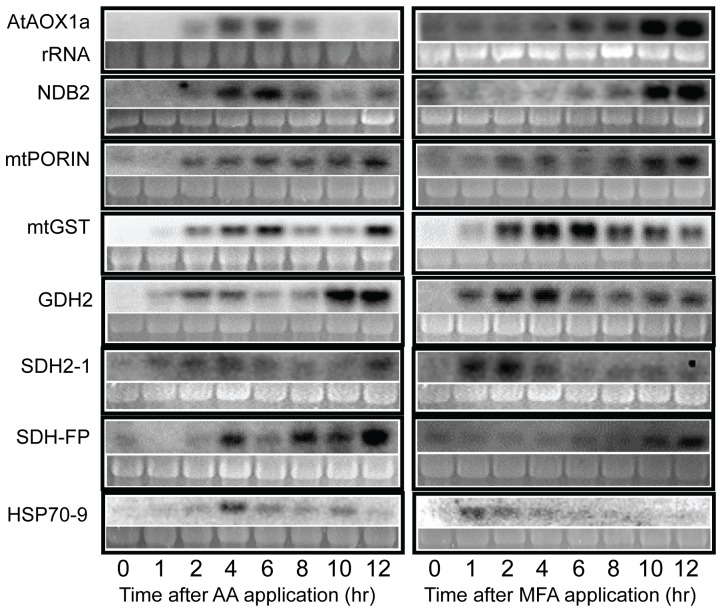
Cytochrome pathway or TCA cycle inhibitions cause differential induction of nuclear genes encoding mitochondrial proteins. Time course experiments in which total RNA was isolated from leaf tissues at indicated times after application of 20 µM AA or 5 mM MFA are shown. RNA (10 µg) was separated by formaldehyde agarose gel electrophoresis and transferred to a charged nylon membrane. Each blot was probed with the digoxigenin-labeled cDNA clone of the gene indicated at left and chemiluminescence was visualized with a cooled CCD camera. RNA loading was assessed by staining of rRNA as shown. Results are representative of 2 or 3 bioreplicates for each gene.

For both AA and MFA treatments, four NEMP genes were induced within 1 h of the beginning of the time course (*mtGST*, *GDH2*, *SDH2-1*, *HSP70-9*; Fig. 3). Following the first hour, four of the eight NEMP genes showed very similar induction patterns when comparing their responses between AA and MFA treatments, although the patterns differed among the genes. *MtPORIN* transcript levels increased at 2 h under both treatments then continued a slow increase throughout the remainder of the time course (Fig. 3). Transcripts for *mtGST*, *GDH2*, and *SDH2-1* had similar times of early peak accumulation for AA and MFA treatment, being between 4–6 h, around 2 h, and between 2–4 h, respectively. With AA treatment, these three genes showed a second peak in transcript accumulation (at 12 h for *mtGST* and *SDH2-1*, and between 10–12 h for *GDH2*; Fig. 3).

Four genes exhibited different transcript accumulation patterns between the two inhibitor treatments. *HSP70-9* transcript accumulation began within 1 h for both treatments, but peaked at 4 h with AA and at 1 h with MFA. Transcripts for *SDH-FP* during AA treatment showed the biphasic induction pattern seen for *mtGST*, *GDH2*, and *SDH2-1*, having peaks at 4 and 12 h. In contrast, for MFA treatment, *SDH-FP* transcript accumulation peaked only at the later time point, 12 h. Within each inhibitor treatment, the bypass pathway genes *AtAOX1a* and *NDB2* were induced together with the same subsequent kinetics (Fig. 3). However, induction for both genes was highest at 6 h after application of AA compared to 10 h after MFA application (based on total counts of hybridized *AtAOX1a* probe for four independent experiments for each treatment, with MFA treatment, there was 15±4% more transcript at the 10 h time point compared to 12 h, and with AA treatment, 19±6% more transcript at the 6 h time point compared to 4 h; Fig. 3). Except for the co-induction of the bypass pathway genes, no other genes were coordinately induced under either inhibitor treatment. As described above, each of the two SDH subunit genes had a different induction pattern, and the stress-related genes had overlapping, but not coordinated, expression patterns.

In control-treated plants, the transcript level for each NEMP gene was barely detectable or undetectable and did not change throughout the time course except for *GDH2*, which showed slight induction at 2 h and 4 h for the AA control and 2 h for the MFA control (not shown). This and the variation in kinetics of induction between the two inhibitors (Fig. 3), demonstrated that circadian rhythm or diurnal regulation could not account for the observed changes in expression of these genes. The low and unchanging control transcript levels are consistent with previously published results [44].

### Leaf Transcriptomes Following AA or MFA Treatment

The times of maximum simultaneous induction of *AtAOX1a* and *NDB2* were used for the microarray experiment time points: 6 h treatment for 20 µM AA and 10 h treatment for 5 mM MFA.

**Figure 4:**
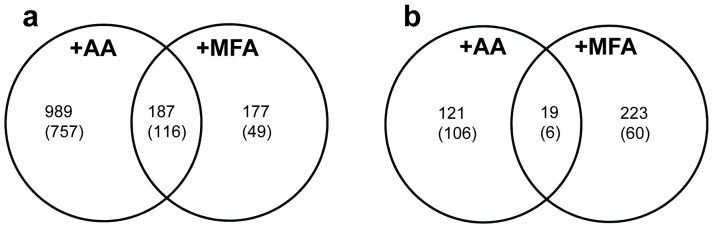
Venn diagram comparing numbers of genes whose expression was affected by 20 µM AA and/or by 5 mM MFA. The total number of genes with q≤0.05 that were up-regulated (a) or down-regulated (b) are shown; In parentheses is shown the number of these genes up-regulated (a) or down-regulated (b) 2-fold or more by each treatment. Note that expression of 9 genes (not shown in the diagram; Resource S1) changed in opposite directions in response to the two inhibitions with all 9 induced by AA but repressed by MFA.

**Figure 5:**
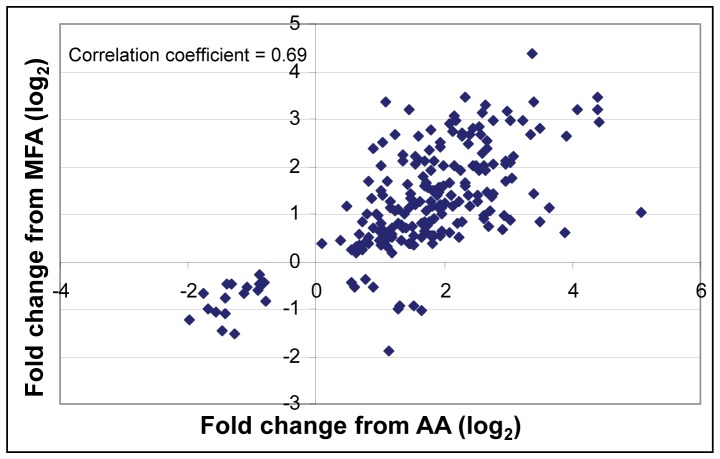
Correlation of gene expression changes from cytochrome pathway inhibition (AA) and TCA cycle inhibition (MFA). Graph of fold change (log_2_) from AA treatment (abscissa) versus fold change (log_2_) from MFA treatment (ordinate) for 215 genes that showed transcript level changes with q≤0.05 for both treatments.

Using the criterion of q≤0.05, 1316 nuclear genes changed in expression in response to AA treatment; 1176 genes showed induction and only 140 genes exhibited decreased expression (Table 1, Fig. 4a and b). MFA treatment resulted in 606 genes with statistically significant altered expression; 364 genes were induced and 242 genes were repressed. Of the 364 induced genes, 187 (51%) were also induced by AA (Fig. 4a). Of 165 genes induced 2-fold or more, 116 (70%) were also induced 2-fold or more by AA (Fig. 4a). Thus, the majority of the genes induced more highly by MFA treatment were also induced more highly by AA treatment. Of the 242 genes down-regulated with MFA, only 19 (8%) were also down-regulated by AA (Fig. 4b). Nine genes showed opposite responses between the two treatments, being up-regulated by AA, but down-regulated by MFA (Resource S1). Two-hundred fifteen genes changed their expression in both data sets (intersections of diagrams in Fig. 4a and b plus the 9 oppositely-regulated genes). A graph of the log-transformed fold changes from AA versus MFA treatments for these genes revealed a good correlation (Fig. 5). Therefore, for most genes whose expression was affected by both inhibitors, their responses were similar at the time points examined.

**Table 1:** Summary of number of Arabidopsis genes with altered transcript accumulation from cytochrome pathway inhibition by AA or TCA cycle inhibition by MFA.

Treatment					Number of Genes				
		q≤0.05			≥2-fold			q≤0.05 & 2-fold	
	Altered	Induced	Repressed	Altered	Induced	Repressed	Altered	Induced	Repressed
AA	1316	1176	140	2663	1341	1322	985	873	112
MFA	606	364	242	515	326	189	231	165	66

The microarray results for AA treatment agreed well with RNA blot analysis of the transcripts of the selected NEMP genes at the corresponding time point. *AtAOX1a*, *NDB2*, *GDH2*, *mtGST*, *SDH-FP* and *HSP70-*9 were significantly induced in the microarray experiment (Table 2; compare to Fig. 3). *SDH2-1* and *mtPORIN* (Fig. 3) were also induced in all AA microarray replicates (data not shown) with q-values of 0.07 and 0.1, respectively (Table 2) due to greater variability. For MFA treatment, *AtAOX1a* and *NDB2* were significantly induced in the microarray experiment (Table 2). *GDH2*, *mtGST*, *mtPORIN*, and *HSP70-9* were also induced in all MFA microarray replicates (data not shown) although variable induction yielded q-values between 0.05 and 0.1 (Table 2). These results are in agreement with the RNA blot results (Fig. 3). MFA microarray data for *SDH-FP* and *SDH2-1* showed highly variable results with some induction in two of the three bioreplicate experiments (data not shown). Consequently, q-values for these two genes were high (Table 2), two instances out of 16 (8 genes with two treatments) where microarray and RNA blot data differed. For any given gene, different methods for measuring transcript quantities can produce different outcomes [37], so some disagreement between the RNA blot data and the microarray data is not surprising.

**Table 2:** Changes in transcript levels for nucleus-encoded mitochondrial protein genes that were altered in expression by AA, MFA, or both treatments.

Gene Locus	AA; q-value	AA; FC (log_2_)	MFA; q-value	MFA; FC (log2)	Gene	Description
At3g50930*#	0.009	2.64	0.03	1.92	BCS1	AAA-type ATPase family protein
At1g48030	0.4	−0.7	0.05	−0.54	MTLPD1	lipoamide DH (mtLPD1); E3 sub.
At3g17240	0.007	0.76	0.1	0.33	MTLPD2	lipoamide DH 2 (mtLPD2); E3 sub.
At4g26910	0.012	0.9	0.5	0.21		dihydrolipoamide succinyltransferase; E2 sub.
At4g26970	0.007	1.46	0.3	0.6	ACO2	aconitate hydratase
At1g72330*∧	0.04	1.67	0.3	0.6	ALAAT2	Ala aminotransferase, put.
At1g17290#	0.05	1.17	0.02	1.12	ALAAT1	Ala aminotransferase, put.
At4g39660	0.04	1.01	0.7	−0.09	AGT2	Ala-glyoxylate aminotransferase, put.
At3g22370#	0.007	3.4	0.003	3.35	AOX1a	alternative oxidase 1a
At1g32350	0.002	2.91	0.1	2.33	AOX1d	alternative oxidase 1d
At4g27585#	0.004	1.93	0.03	0.54		band 7 family prot.
At5g54100	0.003	2.57	0.2	0.47		band 7 family prot.
At3g06850*∧	0.03	0.95	0.09	−0.54	DIN3	branched chain alpha-keto acid DH E2 sub.
At1g10060	0.0006	0.76	0.7	−0.05	BCAT1	branched-chain amino acid transaminase 1
At1g69750#	0.01	1.51	0.03	0.75	COX19	cox19 family protein
At4g10040#	0.03	0.93	0.03	1.01	CYTC-2	cyt. c
At1g22840	0.08	0.93	0.04	0.94	CYTC-1	cyt. c
At3g51790	0.03	0.85	0.4	0.2	ATG1	cyt. c biogenesis/assembly prot., CcmE family
At3g15352	0.01	1.61	0.1	0.4	COX17	cyt. c oxidase copper chaperone related
At4g31500*	0.03	2.3	0.4	−0.22	CYP83B1	cyt. P450 83B1
At1g17745#	6.44E-25	2.07	1.12E-13	0.61	PGDH	D-3-phosphoglycerate DH
At4g34200*	0.01	1.91	0.3	0.49	EDA9	D-3-phosphoglycerate DH
At4g11170*	0.05	1.13	0.4	0.16		disease resistance prot. (TIR-NBS-LRR class)
At1g28210	0.2	0.67	0.003	0.2	ATJ1	strong similarity to mito. DnaJ prot.
At5g25940*∧	0.03	1.28	0.3	0.5		early nodulin-related
At3g08950	0.05	0.93	0.8	0.02	SCO1	electron transport SCO1/SenC family prot.
At5g26030	0.01	1.26	0.2	1.17	FC-1	ferrochelatase I; heme biosynthesis
At5g07440	0.002	2.64	0.08	1.55	GDH2	glutamate DH 2
At3g28850	0.01	0.53	0.25	0.28		glutaredoxin family prot., mito.
At1g02930*	0.02	3.38	0.1	2.09	mtGST	glutathione S-transferase, putative mito.
At3g25610	0.009	2.08	0.06	0.95		haloacid dehalogenase-like hydrolase protein
At4g37910	0.02	2.39	0.06	1.25	HSP70-9	heat shock protein HSP70-9
At4g21870	0.04	0.77	0.7	0.11	HSP26.5	heat shock protein AtHSP26.5, mito.
At5g47590	0.05	0.84	0.8	−0.03		heat shock protein-related
At3g45300*∧	0.04	0.93	0.3	−0.42	IVD	isovaleryl-CoA-DH; Leu catabolism
At1g74360*	0.003	2.82	0.3	0.92		leucine-rich repeat transmembrane prot. kinase, put.
At1g15870	0.6	−0.46	0.04	0.18		mito. glycoprotein family
At1g20350	0.05	0.5	0.6	0.14	TIM17-1	mito. import inner membrane translocase sub.
At1g72750	0.09	0.77	0.03	0.69	TIM23-2	mito. import inner membrane translocase sub.
At3g48850	0.02	1.13	0.07	0.8	PHT3;2	mito. phosphate transporter, putative
At4g24570#	0.04	1.75	0.02	1.57	DIC2	mito. substrate carrier family prot.
At5g27520	0.02	1.41	0.1	0.5		mito. substrate carrier family prot.
At2g22500	0.1	1.06	0.009	1.81	DIC1	mito. substrate carrier family prot.
At5g07320	0.03	0.7	0.8	0.01		mito. substrate carrier family prot.
At5g48970	0.01	0.68	0.7	0.12		mito. substrate carrier family prot.
At4g26180	0.02	0.47	0.6	0.12		mito. substrate carrier family prot.
At5g61810	0.5	−0.48	0.05	0.42	MAC9.1	mito. substrate carrier family prot.
At5g54180	0.3	−0.66	0.05	−0.17		mito. transcription termination factor-related
At4g05020#	0.01	1.35	0.02	2.13	NDB2	external NADH DH
At3g54110	0.01	1.77	0.1	0.57	PUMP	plant uncoupling mito. prot.
At5g15090	0.1	0.79	0.1	0.8	mtPORIN	porin
At3g30775	0.05	1.22	0.5	−0.44	POX	proline oxidase
At5g66760	0.02	1.14	0.4	0.1	SDH-FP	succinate DH flavoprotein sub.
At3g27380	0.07	0.65	0.4	0.29	SDH2-1	succinate DH, iron-sulphur sub.
At5g08300	0.04	1.27	0.1	0.61		succinyl-CoA ligase [GDP-forming] alpha-chain
At2g30720	0.001	1.56	0.4	0.31		thioesterase family prot.
At5g05370	0.05	1.05	0.2	0.65		ubiquinol-cyt. c reductase complex UQ-binding prot., put.

Genes listed either show significant change for one or both treatments or, for some of the genes discussed in the text, are listed regardless of significance. Genes that encode proteins for which there is proteomic data indicating mitochondrial localization or association but not previously annotated as such based on prediction algorithms are indicated by an asterisk [43] and/or a carrot (J.-P.Yu, unpublished). Otherwise, genes were determined to encode mitochondrial proteins based on annotations for the arrays (see ‘Materials and Methods’) or The Arabidopsis Information Resource database. Number symbols indicate the nine genes that were significantly induced by both inhibitor treatments. Fold-change (FC) is the ratio between transcript levels in inhibitor treated plants compared to control treated plants. Ala, alanine; cyt, cytochrome; DH, dehydrogenase; mito., mitochondrial; prot., protein; put., putative; sub., subunit; UQ, ubiquinone.

The microarray data revealed statistically significant expression changes for NEMP genes, more with AA treatment than with MFA treatment. Overall, AA treatment induced 47 NEMP genes, including 6 of the genes used in the RNA analysis, as discussed above. No NEMP genes were down-regulated by AA (Table 2). With MFA treatment, fifteen NEMP genes were induced. Of these, two were genes used in the RNA analysis, *AOX1a* and *NDB2* (see above; Table 2). Two NEMP genes were down-regulated by MFA (Table 2). Of the NEMP genes significantly induced in the microarray experiments with AA or with MFA, nine were in common between the treatments (Table 2).

### Functional Category Analysis of Leaf Transcriptomes

The transcriptome data from each treatment were sorted into functional gene categories (“BINs”) using MapMan. BINs with overall responses statistically significantly different from average (adjusted p<0.05) were identified as described in ‘Materials and Methods.’ Sixty-three and 97 gene categories for the AA and MFA data sets, respectively, were identified out of a total of 704 (main BIN categories and BINs nested within; Resource S2). Twenty-three of these categories showed similar overall induction or repression with both AA and MFA treatments (Resource S2).

**Figure 6:**
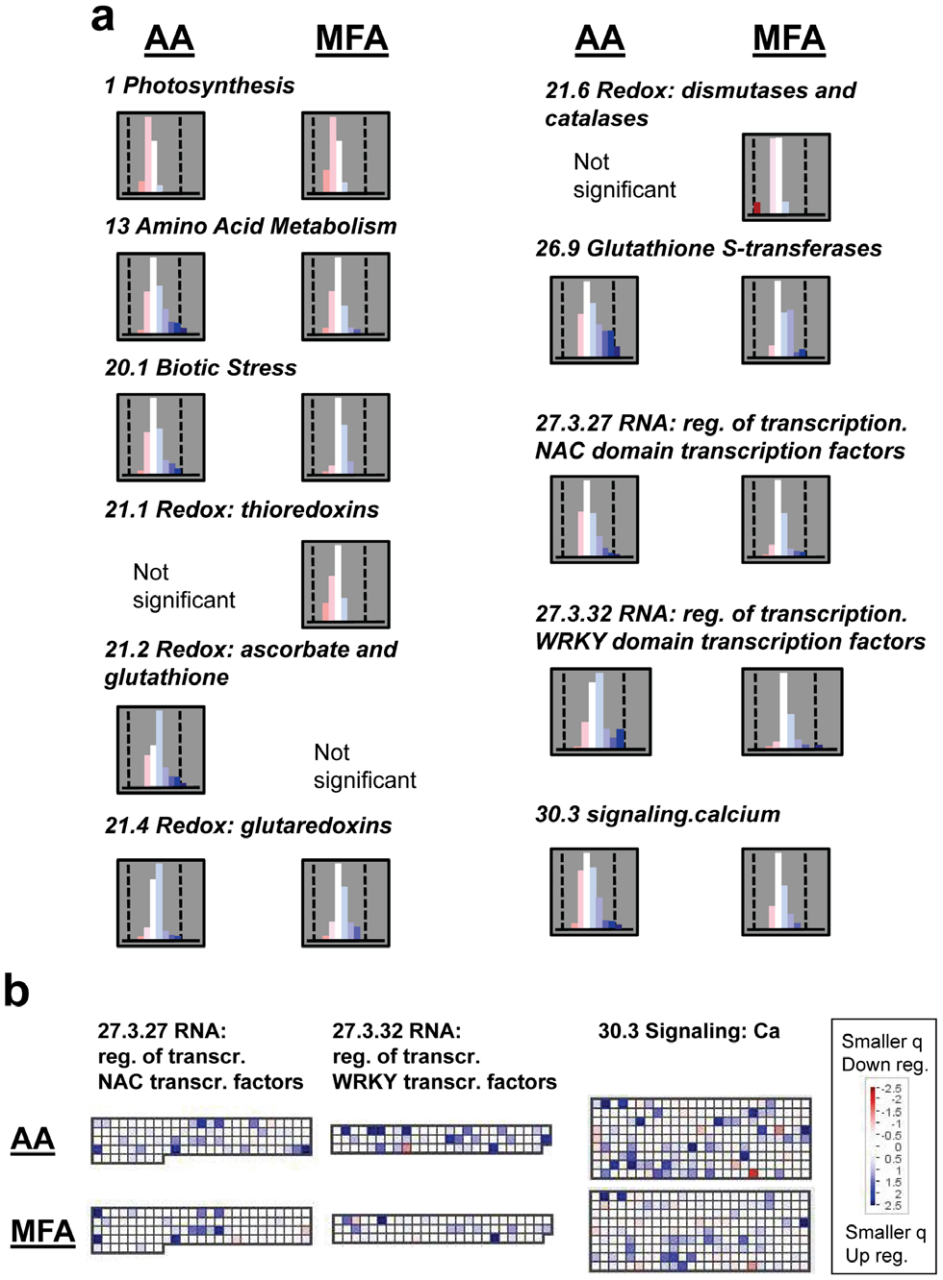
Selected functional gene categories (BINs) generated by MapMan analysis. The q-value data sets, adjusted for transcript directional change, from cytochrome pathway inhibition by AA and TCA cycle inhibition by MFA treatment were used. Red shading indicates genes whose transcript level decreased from treatment; blue shading indicates those whose level increased. a, Gene frequency histograms. The central, white bar of each histogram represents genes with poor statistical significance. The vertical dashed lines are at −2.5 and 2.5 for each histogram. Values less than −2.5 or greater than 2.5 indicate q = 0.0032 or less. Note that the total number of genes in each BIN varies. BIN gene numbers: 1 = 157; 13 = 350; 20.1 = 469; 21.1 = 67; 21.2 = 56; 21.4 = 41; 21.6 = 14; 26.9 = 51; 27.3.27 = 108; 27.3.32 = 74; 30.3 = 225. b, Diagrams from MapMan showing signaling-related functional categories. The logged q-value is shown for each gene (a small square). The correlation between color and intensity and the q-value is shown by the scale. Color scale saturates at 2.5; values less than −1.3 or greater than 1.3 correspond to q-values of 0.05 or less.

Chloroplast-related categories, including those for photosynthesis (BIN 1, with nested BINs for the light reactions and the Calvin cycle) and for protein synthesis in chloroplasts (BIN 29.2.1), were among the most highly statistically significant for both AA and MFA treatments, showing pervasive decreases in transcript abundance (Fig. 6a and Resources S2, S4). For AA only, categories for the mitochondrial TCA cycle and mtETC components (BINs 8.1 and 9, respectively) were affected, with overall increased expression (Resource S2). Also for AA treatment only, cell wall-related categories were affected (BINs 10, 10.1, and 10.6) as was BIN 31.4 for vesicle transport, which showed overall induction (Resource S2).

For both transcriptome data sets, metabolism-related gene categories were identified as responsive. With AA treatment, gene groups encoding enzymes for amino acid synthesis showed overall transcript level increases (BIN 13 and nested BINs, including those for aromatic amino acid and tryptophan synthesis, Fig. 6a and Resources S2, S3, S4), while for MFA treatment the gene groups for amino acid degradation showed overall transcript level decreases (BIN 13 and nested BINs, Fig. 6a and Resources S2, S3, S4). Another group of categories affected oppositely between the two inhibitor treatments were related to protein degradation (BIN 29.5 and nested BINs). These showed overall down-regulation with AA treatment and overall up-regulation with MFA (Resource S2). The MFA treatment data set had additional metabolic gene categories showing overall transcript decreases, including starch synthesis (BIN 2 and nested BINs), minor carbohydrate metabolism (BINs 3 and 3.5), lipid metabolism (BIN 11 and nested BINs), C-1 metabolism (BIN 25), nucleotide metabolism (BIN 23), photorespiration (BIN 1.2), and tetrapyrrole synthesis (BIN 19; Resource S2).

For both AA and MFA treatments, the broad functional category “biotic stress” (BIN 20.1, Fig. 6a and Resources S2, S3, S4) was highly statistically significant, with overall induction. Some categories for genes encoding enzymes to ameliorate effects of oxidative stress were affected in both AA and MFA data sets, showing overall induction: glutaredoxins (BIN 21.4) and glutathione *S*-transferases (BIN 26.9; Fig. 6a and Resources S2, S3, S4). A gene category associated with ascorbate and glutathione metabolism (BIN 21.2) showed overall up-regulation with AA treatment, but not MFA treatment (Fig. 6a and Resources S2, S3, S4).

Most other statistically significant stress-related functional categories were associated with MFA treatment, showing overall repression, including those for dismutases, catalases and thioredoxins (BINs 21.1 and 21.6, Fig. 6a and Resources S2, S3, S4) and a group of genes that respond to abiotic stresses such as drought and salt (BIN 20.2.3, Resource S2). Notably, the general functional category “abiotic stress” (BIN 20.2) was not statistically significant for either of the inhibitor treatments (adjusted p = 0.97 and 0.46 for AA and MFA treatment, respectively; Resource S4).

Functional categories for some signaling-related genes and processes were affected in the same way by both inhibitors. The “signaling” category (BIN 30) and its subgroup specific to calcium signaling (BIN 30.3) as well as categories for genes encoding NAC domain transcription factors (BIN 27.3.27) and WRKY domain transcription factors (BIN 27.3.32) showed overall induction for both AA and MFA treatment (Fig. 6a; Resource S2). Transcript levels of several individual genes of these BINs were similarly affected by both treatments (Fig. 6b; Resource S4). In both data sets, transcripts for genes associated with ethylene-related processes showed increased levels (BINs 17.5 and 17.5.5, Resource S2) and auxin-related gene groups (BINs 17.2 and 17.2.2, Resource S2) were changed also, but transcript levels generally decreased. With MFA only, salicylic acid-related categories (BINs 17.8, 17.8.1; Resource S2) were affected with overall up-regulation.

### Cluster Analysis of Leaf Transcriptomes

**Figure 7:**
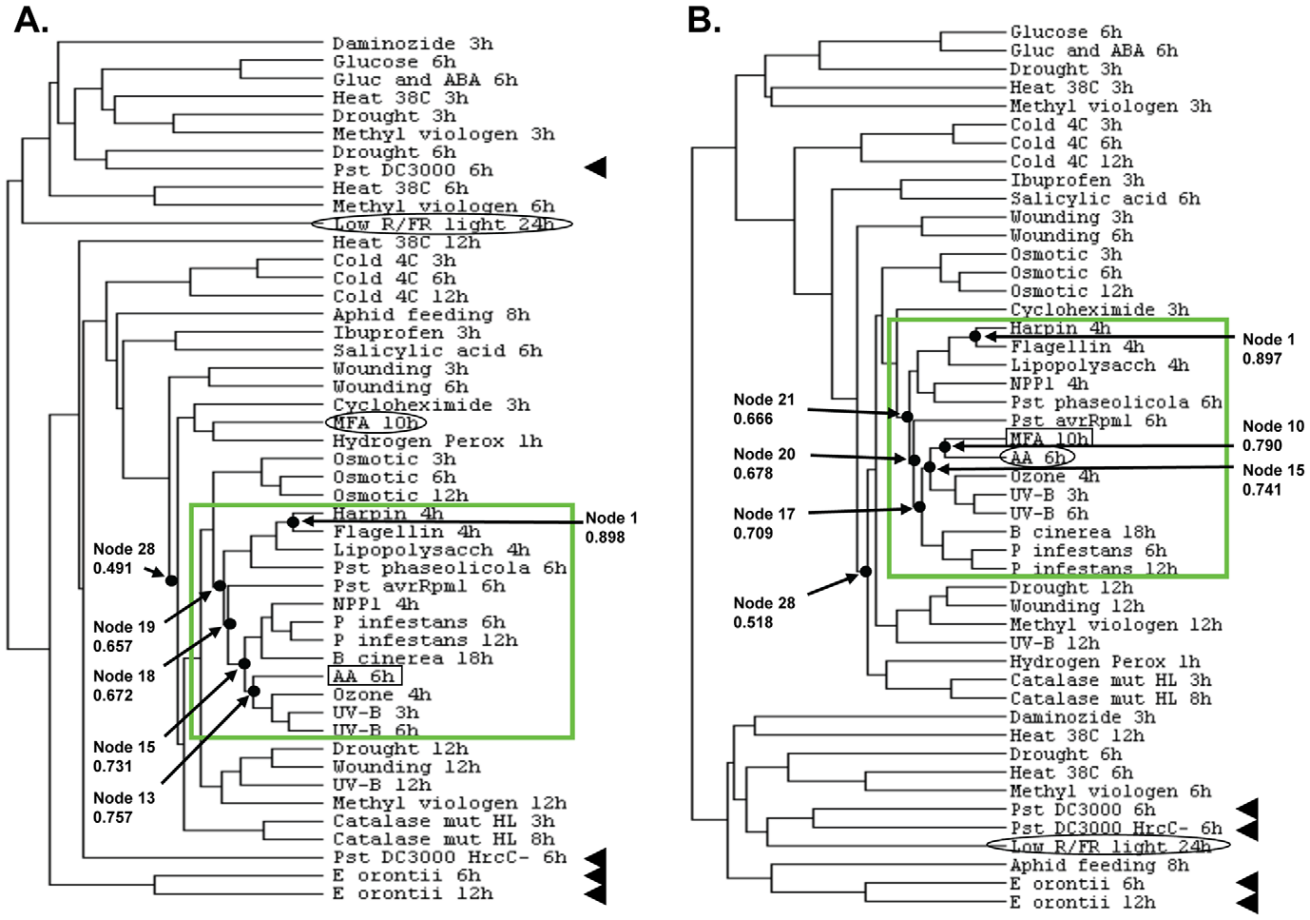
Relationship between AA, MFA, and other stress treatments based on cluster analyses. From public data bases, 46 experiments were chosen that used treatments of leaves or seedlings, and Affymetrix ATH1 arrays. The expression patterns of nuclear genes that were statistically significantly (q≤0.05) altered in expression by AA (a) or that were significantly altered in expression by MFA (b) were compared to their expression patterns in the transcriptomes resulting from the 46 stress treatments (Resource S5) and from the other inhibitor treatment. Their expression ratios in treatment versus control were compared with those from AA or MFA treatment using Cluster (Hierarchical Clustering/Average Linkage Clustering). The resulting array clusters were visualized using TreeView. The query gene set (i.e., transcriptome) for each inhibitor is indicated by a box in a and b, while the non-query inhibitor gene set is circled. A photomorphogenesis experiment transcript subset that served as an outgroup is circled in a and b. Pathogen and pathogen-related treatments clustering near AA and MFA are delimited by a green box; pathogen treatments elsewhere in the tree are designated with arrow heads to the right. Correlation coefficients for the tree nodes (Resource S6) range, left to right, in a. from −0.112 to 0.898 and in b. from −0.105 to 0.897. Numbers of designated nodes and their correlation coefficients are shown in the figures.

In order to determine whether the genes whose transcript levels were affected by AA or MFA treatment are affected similarly by biotic, abiotic, or oxidative stresses imposed on aerial tissue, two cluster analyses were performed. The genes with transcript abundance changing in response to either AA or MFA were used as separate query sets and compared to the responses of these same genes (termed here “transcript subset”) in 47 other experiments in which a stress was applied (Resource S5), including stresses representative of the biotic, abiotic, and oxidative categories and the other inhibitor experiment; the MFA experiment was included in the cluster analysis using the AA-affected transcript subset and vice versa. Two trees (Fig. 7) were derived from correlation coefficients (Resource S6) for the cluster nodes. The corresponding heat maps for the trees are shown in Resources S7 to S15. A photomorphogenesis experiment, low red/far red light, served as an out-group (circled in Fig. 7a and b). The profile of the transcript subset from this light treatment was not positively correlated with the gene transcript abundance changes in response to AA or MFA. The transcript expression pattern resulting from AA treatment, when used as the query set, clustered at node 13 (correlation coefficient 0.76) with transcript subsets from ozone treatment, and two of the three UV-B treatments. The next-closest node, 15 (correlation coefficient 0.73), joins with fungal (*Botrytis cinerea*) and oomycete (*Phytophthora infestans*) pathogens and the *P. infestans* elicitor, NPP1. Subsequent nodes join with the bacterial pathogens *Pseudomonas syringae* avrRpm1 (node 18, correlation coefficient 0.67) and 6 h *P. syringae* phaseolicola, and the bacterial elicitors flagellin [45], lipopolysaccharide [46], and harpin, which disrupts mitochondria [47] (node 19, correlation coefficient 0.66; Fig. 7a). The AA transcriptome overlaps the MFA transcriptome by less than 16% (Fig. 4) which probably accounts for their low correlation in this tree (node 28, correlation coefficient 0.49; Fig. 7a). When genes whose expression was affected by MFA treatment were used as the query, essentially the same clustering with respect to ozone, UV-B, pathogens and pathogen elicitors relative to AA was observed (see Fig. 7b for nodes and correlation coefficients) with two exceptions. For one, in this tree, MFA closely correlated with AA, at node 10 (correlation coefficient 0.79), likely due to the MFA transcriptome having a large percent of genes also affected by AA treatment (Figs. 4 & 5). For the other, NPP1 treatment clustered with *P. syringae* phaseolicola, close to the bacterial elicitors (Fig. 7b).

Thus, in both trees, there was an enrichment of pathogen and pathogen-related treatments in the nodes closest to AA and MFA, and, except for ozone and UV-B, abiotic stresses occurred at more weakly correlated nodes. However, four pathogen-treatment experiments did not cluster close to AA, MFA, or the other pathogen and pathogen-related treatments. The transcript subsets from two *P. syringae* treatments, DC3000 6 h and HrcC- 6 h, and *Erysiphe orontii* 6 and 12 h treatments were in nodes distant from the AA and MFA treatment data, similar to the light treatment outgroup (Fig. 7a and b).

## Discussion

We applied inhibitors of two major components of mitochondrial respiration, the cytochrome pathway of the mtETC and the TCA cycle, to intact plant leaves in order to disrupt mitochondrial steady-state function, thereby triggering a mitochondrial response and MRR. Experimental treatments of leaves were performed in the dark to maximize effects of the inhibitors on mitochondria, while minimizing potential non-mitochondrial effects. In chloroplasts, antimycin A inhibits one of two types of cyclic electron transfer around PSI [48], [49] which is inactive in the dark [49], [50]. In leaves in the dark, TCA cycle activity will be high compared to its non-cyclical, diminished activity in the light [51]. Further, although MFA inhibits the glyoxylate cycle [52], this cycle appears to be inactive in light-grown Arabidopsis leaves and is not induced during darkness [53], [54]. Thus, to our knowledge, AA and MFA inhibit respiration by defined and specific mechanisms under the conditions used in our experiments, providing means to disrupt specific mitochondrial functions.

### Time Course of NEMP Gene Transcript Accumulation With Different ROS Levels

The two inhibitor treatments resulted in very different ROS production. Using DCF fluorescence, we measured an increase in ROS in Arabidopsis leaves during AA treatment of either intact or excised leaves, as previously observed for suspension culture cells of Arabidopsis, tobacco, and soybean (see Introduction). For the excised leaves, ROS production was approximately equivalent in the presence of either 10 or 25 µM AA (Fig. 1 a and d) indicating that cytochrome respiratory pathway inhibition was saturated at 10 µM AA. The higher level of ROS production seen with the excised leaves, compared to the intact leaves (compare Figs. 1 and 2), may be due to more complete inhibitor penetration into the tissue combined with stresses imposed by excision and soaking of the leaves. With MFA treatment, we found no ROS increase in leaves from treated intact plants or when excised leaves were incubated in MFA solution (Figs. 1 and 2). Similarly, MFA treatment did not increase ROS production by Arabidopsis suspension culture cells [35]. However, ROS production by tobacco cells, also measured by DCF fluorescence, was as great with MFA treatment as it was with AA treatment [8], [24], suggesting that species respond differently to MFA inhibition.

To our knowledge, only tobacco and Arabidopsis culture cells and Arabidopsis leaves (this study) have been tested for the effect of MFA on tissue ROS level, leaving open the question whether there is a typical plant tissue response to MFA with respect to ROS production. The events leading to increased ROS in tobacco cells in the presence of MFA have not been elucidated. TCA cycle inhibition by MFA presumably would serve to decrease the supply of reductant for the mtETC, creating a relatively oxidized state rather than the over-reduction that occurs with AA inhibition. However, low levels of reductant could curtail regeneration of ROS buffer systems in the mitochondria, and ultimately lead to increased mtROS. Differing activities in Arabidopsis and tobacco of the GABA shunt, a NADH-producing partial bypass pathway for the TCA cycle linked to decreased tissue ROS production [55], is one possible explanation for the observed difference in ROS levels in the presence of MFA.

To determine if accumulation of transcripts for mitochondrial proteins was affected during mitochondrial inhibition, whether or not ROS production increased, expression of eight NEMP genes was followed over a 12 h time course. Regardless of ROS level, transcripts for four of the eight NEMP genes began to accumulate within 1 h during both mtETC inhibition and TCA cycle inhibition, indicating that MRR and subsequent expression changes occurred quickly in response to the inhibitions.

For the early part of the time course, through 6–8 h, four genes showed similar transcript accumulation patterns for both treatments (*mtPORIN*, *mtGST*, *GDH2*, and *SDH2-1*) consistent with mtROS-independent MRR pathway(s) operating during both mtETC inhibition and TCA cycle inhibition. Four genes showed different accumulation patterns between the treatments (*HSP70-9*, *SDH-FP*, *AOX1a*, and *NDB2*) and three of the first group of genes (*mtGST*, *GDH2*, and *SDH2-1*) showed a second late accumulation peak with only AA treatment. This pattern variety, evident across and within the groups of selected genes, could result from mtROS-dependent and –independent signaling. For example, the time of maximum accumulation for the transcript pair of *AOX1a* and *NDB2* was separated by 4 h between the AA and MFA treatments. During AA inhibition, elevated mtROS could act as a signal for increased *AtAOX1a* expression as previously reported [8]. However, because MFA treatment did cause *AtAOX1a* and *NDB2* induction with a pattern different from AA, a mtROS-independent MRR pathway also appears able to induce these genes. Recent indirect evidence indicates that malonate inhibition of succinate dehydrogenase, a component of both the mtETC and the TCA cycle, does not increase mtROS production in Arabidopsis seedlings [4], yet it does increase *AOX1a* and *NDB2* transcript abundance in Arabidopsis culture cells [19]. Subject to further investigation, malonate may be another mitochondrial inhibitor that triggers MRR without mtROS, at least in Arabidopsis.

Because treatment of attached Arabidopsis leaves with MFA did not increase tissue ROS levels, unlike in previous studies, we could compare MRR signaling subsequent to known mitochondrial perturbations under conditions of different ROS production. During AA inhibition, ROS production by mitochondria has been observed directly [7], [56], evidence that ROS involved in MRR signaling pathways triggered by AA are specifically mtROS [7], [8]. Absence of measurable ROS with MFA treatment in our study suggests that mtROS levels were not increased and MRR was independent of mtROS during this treatment. However, while tissue ROS levels do reflect intracellular ROS amounts [57], small concentration transients in mtROS or other ROS pools could have occurred that were not detected by our tissue-level measurements, a common limitation among most MRR studies [8], [23], [24], [36]. In order to verify the presence of a truly mtROS-independent MRR pathway(s), subcellular monitoring of ROS production by mitochondria during these transient disruptions will be necessary.

### Transcriptome Consequences of MRR and Mitochondrial Metabolic Restriction

The effects of mitochondrial inhibition and MRR on expression of certain NEMP genes have been well-studied. Less studied is the extent to which mitochondrial inhibition, encompassing signaling and metabolic effects, impacts expression of nuclear genes in general. We obtained a snapshot of the plant transcriptome during mitochondrial inhibition in the presence or absence of elevated ROS levels through a microarray experiment. We chose a time point for each treatment when *AOX1a* and *NDB2* transcripts were at maximum abundance. Increased abundance of protein or gene transcripts for AOX in plant tissue is considered a stress indicator [58], but AOX can decrease the formation of mtROS from the mtETC and, with NDH, can process excess cellular reductant [18]. Because these mtETC bypass proteins may modulate oxidative stress and stress signaling [9], [58], [59] and help to maintain metabolic homeostasis [60], their transcripts together act as a landmark of a transcriptome-level response to stress that will help to bring about recovery. We focused on determining the whole transcriptome response concurrent with the maximum changes in *AOX1a* and *NDB2* expression in order to better understand how cells adjust to mitochondrial perturbations in coordination with this change in the mtETC.

Many changes, relative to control, were observed in the transcriptomes of AA- and MFA-treated leaves. Some of these changes were shared between the transcriptomes, especially for the most highly induced genes (2-fold or higher) where 70% of the genes induced by MFA were also induced by AA. While most of the affected genes were not NEMP genes, a number of NEMP genes did respond to the inhibitor treatments, with some being induced by both. Although the inhibitors have distinct mitochondrial sites of action, the similarities in transcriptome responses may reflect ongoing common MRR signaling, as suggested by the signaling-related functional categories (WRKY and NAC domain transcription factors and calcium signaling) affected by both treatments. The transcriptomes may also reflect a common metabolic response to restriction of mitochondrial function caused by the inhibitors.

Some broad features also differed between the responses with the transcriptome resulting from cytochrome pathway inhibition consisting of mostly up-regulated genes (89%), and that resulting from TCA cycle inhibition showing relatively more down-regulation (40%), including functional gene categories for starch synthesis, photorespiration, and C1 metabolism. The transcriptome of rotenone-inhibited Arabidopsis culture cells also included down-regulation of these same functional categories [17], and, like with MFA, *AOX1a* and *NDB2* were highly induced at a later time point (12 h). Metabolic analysis showed that the TCA cycle had slowed in the rotenone-treated cells [17] providing a basis for similarity between the transcriptomes resulting from MFA and rotenone treatments. Although AA inhibition of the mtETC blocks two of three phosphorylation sites with a decrease in ATP as a consequence [8], [61], this may be less restrictive metabolically than TCA cycle inhibition by MFA. During AA inhibition, the TCA cycle can operate when the alternative respiratory pathway is present, preserving basic mitochondrial function [61].

A highly-significant functional gene category with overall down-regulation for all transcriptomes following mitochondrial inhibition (AA, MFA, this study; rotenone, [17]) is for the light reactions of photosynthesis. This result underscores the close signaling and metabolic relationship between chloroplasts and mitochondria (e.g., [27], [62], [63]).

MapMan and cluster analyses provided insight into how the transcriptome as a whole responded to the mitochondrial inhibitions compared to other stresses. Glutaredoxins and glutathione *S*-transferases functional categories showed overall induction for both AA and MFA treatments. For AA treatment, the ascorbate and glutathione functional category, whose 56 genes primarily encode enzymes for elimination of ROS, showed overall induction. These outcomes suggest some genomic response to oxidative conditions may have occurred, particularly during AA treatment. However, with both inhibitor treatments, the general “abiotic stress” category was not significant, and, for MFA treatment, the subset abiotic stress category for drought and salt, and other categories related to oxidative stress (dismutases, catalases, and thioredoxins) were statistically significant but with over-all down-regulation. In the cluster analysis, most of the abiotic stress (cold, heat, and drought) and oxidative stress-related (methyl viologen, catalase mutant in high light, and hydrogen peroxide) transcript subsets were weakly correlated with the AA and MFA treatment query sets. This clustering outcome suggests that the genes typically affected by abiotic and oxidative stresses were affected to a different degree, or not at all, during treatment with the two mitochondrial inhibitors.

Thus, MapMan and cluster analysis each indicated that the Arabidopsis leaf transcriptome did not strongly respond as though the leaves had been subjected to an abiotic or oxidative stress, even with increased ROS production during AA treatment. This result is consistent with observed differences between effects of AA and H_2_O_2_ on gene expression in leaves [22], and a growing body of evidence suggesting that the origin and type of ROS [8], [19], [64]–[67] as well as the amount of ROS from a given subcellular origin [68] are factors distinguished by plant cells, leading to distinct gene expression responses.

Rather than an association with abiotic or oxidative stress, the cluster analysis showed that the genes affected by the mitochondrial inhibitors were most similarly affected under the biotic stress conditions of pathogen challenge and bacterial elicitor exposure (with the exceptions of ozone and UV-B; see below). Also, MapMan analysis showed “biotic stress” to be one of the most statistically significant functional gene categories for both AA and MFA treatments. This category includes genes encoding disease-resistance proteins with TIR, TIR-NBS, TIR-NBS-LRR, and CC-NBS-LRR domain signatures and genes encoding proteases and avirulence-responsive proteins.

Other affected functional categories for AA and MFA showed transcript changes previously observed with biotic stress, specifically pathogen attack. Photosynthesis rates decrease [69], accompanied by down-regulation of transcripts for associated photosynthesis genes, in a variety of plant-pathogen interactions [70]–[73]. As noted above, AA and MFA treatments resulted in striking down-regulation of genes related to photosynthesis. Repression of auxin signaling, which the functional category analysis suggests occurred with AA and MFA treatment, appears to be an important protective plant response to pathogens [74], [75]. Increased expression of genes for ethylene synthesis is observed with pathogen challenge [76], [77] and ethylene functional categories were significant and up-regulated overall with AA and MFA treatment. Lastly, for MFA-treated leaves, functional categories for salicylic acid, well-known for its role in plant defense [78] were affected, with most genes up-regulated.

Transcriptome changes suggesting increases in the amino acid pool of the leaves were also consistent with plant responses to pathogens, although the changes differed between the two inhibitor treatments. For AA-treated leaves, functional category analysis indicated that expression of genes encoding enzymes involved in aromatic amino acid synthesis, including tryptophan synthesis, increased. Similar expression changes have been observed upon bacterial infection of Arabidopsis leaves [77]. These amino acids are precursors of defense compounds including phenylpropanoids, alkaloids, flavonoids [79] and the anti-pathogenic phytoalexin, camalexin [80]. Functional categories for MFA-treated leaves also indicated possibly increased amino acid pools, but accomplished through increased protein degradation and repression of amino acid breakdown pathways. These changes could reflect an attempt, with the TCA cycle restricted, to funnel amino acids from proteins into anti-pathogen secondary metabolite synthesis or to mobilize nitrogen sources away from a perceived infection site [69].

With AA treatment only, the TCA cycle and mitochondrial electron transport functional categories were significantly affected, showing overall up-regulation, consistent with the increased respiration that accompanies the resistance response [69], [81]. The AA-treated leaf transcriptome also had significant functional categories for cell wall processes and for vesicle transport, allowing for increased secretory activity which provides for the movement of anti-pathogen molecules to the cell surface [82].

Further support for a link between MRR and pathogen stress response comes from the cluster analysis. The transcript subsets for *P. syringae* DC3000 HrcC^−^ and DC3000 treatments did not correlate with the AA- and MFA-treatment transcriptomes or the other pathogen and pathogen-related treatments. This is because these strains, unlike *P. syringae* phaseolicola and avrRpm1, do not express avirulence factors recognized by Arabidopsis, and therefore do not trigger the plant's pathogen resistance response [83] to affect expression levels of the types of genes found, for example, in the biotic stress functional category. The *Erysiphe orontii* treatment transcript subsets also were not correlated with AA and MFA. *Erysiphe* is an obligate biotroph, compared to the fungal necrotroph *Botrytis* and the oomycete facultative necrotroph *Phytophthora* [84]. The different pathogenesis modes between biotrophs and necrotrophs may account for *Botrytis* and *Phytophthora* triggering plant transcript subset profiles that correlate closely with the AA- and MFA-treatment transcriptomes while *Erysiphe* does not.

Of the two abiotic stresses that clustered with AA and MFA, ozone, when applied to tobacco leaves, rapidly inhibits the mitochondrial cytochrome oxidase pathway [85], making it likely that the same genes affected by the cytochrome pathway inhibitor, AA, would also be affected by ozone. For UV-B, algal cell mitochondrial morphology is commonly disrupted upon exposure to it [86] and low levels of UV-B cause rapid loss of mitochondrial membrane potential [87], while higher doses lead to mitochondrial fragmentation and swelling [88], in human cells. The clustering we observed here of UV-B with the mitochondrial inhibitors and ozone suggests that UV-B may have specific effects on plant mitochondria, as well, but this requires further investigation.

In total, with AA treatment, the transcriptome contains more elements of the transcriptome of a pathogen-challenged plant than with MFA treatment. This may be due to the elevated ROS levels from AA treatment signaling a more complete response. On the other hand, the MFA-treatment transcriptome pattern suggests that at least some of the response to pathogens can occur without elevated ROS. The Complex II mutant, *dsr1*, cannot signal for induction of a reporter gene under stress conditions because it is unable to increase mtROS production. This mutant is more susceptible to a fungal pathogen and a virulent bacterial pathogen, but shows no altered resistance to avirulant bacteria, further suggesting the possibility that some resistance pathways may operate without elevation in mtROS levels [4].

Changes in the mitochondrial proteome [89] and leaf respiration [81] occur early during pathogen infection. Our data suggest that in the short term, steady-state disruption by TCA cycle inhibition and mtETC inhibition especially may partially mimic mitochondrial disruptions caused by pathogens, with MRR and mitochondrial processes contributing to the responses of plants to pathogens. The observation that the content of putrescine, a polyamine that may be involved in stress response and protect against cell death [90], [91], was elevated in AA-treated leaves (C.C. Subbaiah & D.M. Rhoads, unpublished data) is consistent with this possibility. Other inhibitor-based studies using AA treatment have suggested a link between mitochondrial perturbation and response to pathogens but were based on transcript profiles of a few genes [8], [38], [92]. For the previous analysis of a large number of genes [39], transcriptome changes in common with many abiotic stresses, as well as with virus infection, were found, but these changes could not be distinguished from leaf excision or submergence. Mitochondria may be important for fighting and surviving infection by re-routing metabolism [34], [41] in addition to their more commonly recognized role in programmed cell death [2], [9].

### Conclusions

The mtETC inhibitor AA increased ROS production by Arabidopsis leaves, but the TCA cycle inhibitor MFA had no detectable effect on ROS levels. Despite the clear difference in the observed ROS levels, both mitochondrial perturbations quickly resulted in increased NEMP gene expression, most likely to begin mitochondrial adjustments to the restrictions. Time course expression patterns of several genes were the same with both inhibitors, while patterns for others differed, including the coordinately regulated genes for enzymes of the mtETC bypass pathway. These transcript accumulation patterns show that large increases in ROS are not needed for MRR, and are consistent with the presence of mtROS-dependent and –independent MRR signaling pathways. However, no increase in ROS production by mitochondria specifically will need to be shown to verify that mtROS-independent MRR occurs. Transcriptome analysis showed that disruption of the mitochondria by either inhibitor affected transcript abundance for many nuclear genes. With AA treatment and increased ROS, the transcript profiles suggested some genomic response to oxidative stress. However, the gene expression changes observed for both mitochondrial perturbations, despite their different sites of action, corresponded most closely to expression changes of the same genes in response to biotic stresses. Thus, the possibility that restriction of mitochondrial function and MRR are involved in biotic stress responses is supported by our observations.

## Materials and Methods

### Plant Growth and Treatments

*Arabidopsis thaliana* Col-0 was grown, with separate plantings for microarray and RNA blot experiments and leaf ROS measurements, in Sunshine Mix #1 (Jero, Inc., Waddell, AZ, USA) soil at 21°C during 16 h of light of 100 µmol m^−2^ s^−1^ and 18°C during the 8 h dark period. Plants were fertilized as described [31]. Initial inhibitor treatment of plants used for microarray experiments, RNA blot time-course experiments, and intact leaf ROS measurements was at 20 days after germination. In each of these experiments, for AA treatments, 20 µM AA (Sigma A-8674; Sigma-Aldrich Corp., St. Louis, MO, USA) in 0.01% Tween 20, and for MFA treatments, 5 mM MFA in 0.01% Tween 20, were each sprayed onto plants as described [31]. For both inhibitor treated and control treated (sprayed with appropriate solutions lacking inhibitors) samples, after an initial 3 h in the light, plants were moved to the dark for 2 h prior to spraying. All plants were incubated in the dark during treatments. Plants grown in soil and treated with AA or MFA in the dark were able to survive treatment with no observable short-term (up to 24 h) effects and all plants not used for RNA isolation survived the treatments long-term and flowered.

### Measurement of ROS

Leaf ROS production, H_2_O_2_ and other peroxides, specifically, was measured using H_2_DCFDA (Molecular Probes, Eugene, OR, USA). In a set of experiments to determine ROS production under conditions used to obtain the time course RNA blot data and the microarray data, intact plants in soil at 20 days after germination were control treated or treated with inhibitors exactly as described above, including the incubation in the dark prior to inhibitor treatments. At the specified time points, leaves were excised and incubated for 30 min in H_2_DCFDA at a final concentration of 20 µM in 10% Murashige and Skoog basal salt mix [93] plus 0.1% Tween-20 (6–8 leaves in 3 mL of medium) at about 25°C in the dark. Three separate batches of leaves (i.e., from three different pots) were incubated for each treatment at each time point. At the end of 30 min, an aliquot from each batch was taken to quantify fluorescence from DCF in a Perkin Elmer LS-5B Luminescence Spectrophotometer with excitation set at 488 nm and the emission set at 525 nm and leaf dry weights were used to normalize the data.

In another set of experiments, leaves were excised and transfered to 10% Murashige and Skoog basal salt mix [93] plus 0.1% Tween-20 (5–8 leaves in 10 mL of medium). H_2_DCFDA was added to a final concentration of 20 µM, followed by a 30 min pre-incubation at about 25°C in the dark for uptake. For experiments for Fig. 1d, plants were incubated in the dark for 2 hr prior to excision. Samples were then either control-treated (incubated in solution lacking inhibitors) or AA was added to a final concentration of 10 or 25 µM, MFA was added to 5 mM, or menadione was added to 100 µM or 500 µM and samples were incubated at about 25°C in the dark for the specified times. Fluorescence was visualized at various time points using a Kodak Image Station 2000 MM with excitation and emission wavelength set at 488 nm and 525 nm, respectively. Quantification was done by reading fluorescence from DCF in aliqouots taken from the medium using a SPECTRAmax M2 spectrofluorometer (Molecular Devices, Sunnyvale, CA, USA) for data for Fig. 1a, or a SPEX FluoroMax™ spectrofluorometer (SPEX Industries, Inc., Edison, NJ, USA) for data for Fig. 1d with the excitation set at 488 nm and the emission set at 525 nm. Leaf wet weights were used to normalize the data. DAB was used for qualitative, visual detection of H_2_O_2_ following the procedure of Thordal-Christensen et al. [94].

### DNA Manipulations and Sequencing

All plasmid and DNA fragment manipulations were done according to standard protocols [95]. DNA fragment purification was done using the Geneclean Kit (MP Biomedicals, Irvine, CA, USA) according to the protocol of the manufacturer. Restriction endonucleases were from Promega (Madison, WI, USA) or Fermentas, Inc. (Hanover, MD, USA) and digestions were done according to the protocols of the manufacturers. DNA sequencing to confirm cDNA clone identities was done by the DNA Laboratory at Arizona State University using a 3730 Capillary Array Sequencer (Applied Biosystems, Foster City, CA, USA).

### RNA Isolation and Blot Analyses

Total RNA was isolated from plants (typically 0.5–1 g of rosette tissue) using TRIzol according to the protocol of the manufacturer (Invitrogen, Carlsbad, CA, USA). RNA was separated by agarose-formaldehyde gel electrophoresis [95]. Fluorescence from ethidium bromide stained ribosomal RNA was recorded using a UVP Gel Analysis System and the LabWorks Imaging and Analysis program (UVP, Inc., Upland, CA, USA) according to the instructions of the manufacturer. RNA was then blotted to nylon membrane according to standard protocols [95]. DNA probes were labeled with Digoxigenin HighPrime Labeling and Detection Kit and used for hybridizations according to the protocols of the manufacturer (Roche Diagnostics Corporation, Indianapolis, IN, USA). For detection of *AtAOX1a* transcript, a labeled fragment from an *AtAOX1a* cDNA clone [96] was used. For other genes, labeled inserts from the following cDNA clones were used: *NDB2*, U12861; *mtPORIN*, 124F4; *AtHSP70-9*, U15562; *mtGST*, U13302; *GDH2*, 248A23; *SDH2-1*, U09779; *SDH-FP*, U13004. Hybridization was visualized using the NightOwl cooled CCD camera system and the WinLight 32 analysis software (Berthold Technologies USA LLC, Oak Ridge, TN, USA).

### RNA Isolation, Treatment and Target Labeling for Microarray Experiments

Total RNA was initially extracted from plants using TRIzol as described above and treated with TURBO-DNase (TURBO DNA-*free* kit, Ambion, Austin, TX, USA) according to the manufacturer's instructions. RNA concentrations were determined spectrophotometrically and integrity was checked on a 1.2% agarose-formaldehyde gel according to Sambrook et al. [95]. Total RNA was further purified using an RNeasy MinElute Cleanup Kit (Qiagen, Valencia, CA, USA). Labeled aRNA was produced using Amino Allyl MessageAmp aRNA Amplification Kit according to the protocols of the manufacturer (Ambion, Austin, TX, USA). In brief, 2.0 µg of total RNA was used for first-strand cDNA synthesis. Second-strand cDNA was produced and double-strand cDNA was used in an *in vitro* transcription reaction to generate amino allyl modified aRNA, 5–10 µg of which was used for coupling to either Cy3 or Cy5 dyes (Amersham Biosciences, Piscataway, NJ, USA). After coupling at 25°C for 60 min, the reaction was quenched with hydroxylamine. The Cy3- or Cy5-labeled target was purified using an RNeasy MinElute Cleanup Kit (Qiagen, Valencia, CA, USA).

### Microarray Preparation, Hybridization, Washing and Imaging

Microarrays used for these experiments were ATv3 x.y.z microarrays produced using the Operon-Qiagen Arabidopsis Genome Array Ready Oligo Set (AROS) version 3.0, which represents >26,000 loci (Qiagen, Valencia, CA, USA). Microarrays were prepared, hybridized and washed following procedures based on the recommendations of the manufacturer (Oligonucleotide Microarray Facility, University of Arizona, Tucson, AZ, USA). Prior to hybridizations, microarrays were rehydrated by exposure to water vapor produced by a 45°C water bath for 5–10 s and then snap dried on a 65°C heating block for 5 s. This cycle was repeated three times with cooling to about 25°C between each cycle. DNA was cross-linked to the microarrays by exposure to 180 mJ UV-radiation using a Stratalinker (Stratagene, La Jolla, CA, USA). Hybridization in each case was with aRNA made using RNA isolated from inhibitor-treated tissue and labeled with Cy3 or Cy5 and aRNA made using RNA isolated from control treated tissue and labeled with the complementary dye (Cy5 or Cy3, respectively). Microarrays were then washed in 1% SDS for 5 min at about 25°C on a shaker, dipped several times into sterile diethyl pyrocarbonate-treated water, and immediately dipped in and washed with 100% ethanol with shaking at 25°C for 3 min. Finally, microarrays were dried by centrifugation at 1000 g for 3 min in slide holders in a table-top centrifuge and used for hybridization. Hybridization mixture (60 µL) consisted of 48 or 96 pmol of dye (which corresponded to about 0.3–0.6 µg of labeled aRNA), 3.6 µL of liquid block (GE Healthcare Bio-Sciences Corp., Piscataway, NJ, USA), 2× SSC, and 0.08% SDS (w/v). The hybridization mixture was incubated in a boiling water bath for 2 min and transferred to ice. A raised cover slip (Fisher Scientific, Pittsburgh, PA, USA) was placed over each microarray and hybridization mixture was applied to one end of this open-ended chamber. The microarray was immediately placed in a hybridization chamber and incubated overnight (8–12 h) at 55°C. After hybridization, microarrays were washed successively for 5 min in 2× SSC and 0.5% (w/v) SDS at 55°C, followed by 0.5× SSC and then 0.05× SSC, both at about 25°C. The microarray was dried by centrifugation at 1000 g for 3 min and scanned using a G2565BA Microarray Scanner System (Agilent, Palo Alto, CA, USA). Quantitative data were extracted from image files of microarrays and further analyzed using GenePix Pro 6.0 software (Molecular Devices, Sunneyvale, CA, USA) and the appropriate GenePix Array List file from the Oligonucleotide Microarray Facility.

### Statistical Analysis of Microarray Data

Three biological replicate experiments (each including treatment and control) with dye-swapping (6 microarrays) and a duplicate slide of one dye condition for each of two of the biological replicates (for 8 total microarrays) were performed and used in the analyses for AA-treated versus control samples. Three biological replicate experiments (each including treatment and control) with dye-swapping (6 microarrays) were performed and used in analyses for MFA-treated versus control samples. For analyses of the AA-treated or MFA-treated versus control samples, differences in transcript levels were determined by a two-step mixed model analysis of variance (ANOVA) procedure [97], [98]. Data from GenePix software files for each array were log_2_-transformed. In the first ANOVA step of the mixed model, these values were scaled and normalized. In the second ANOVA step, these data were used to determine statistically significant differences in gene transcript levels between treated and control samples. The ANOVA steps were performed in SAS version 9 (SAS Institute, Cary, NC, USA). A false discovery rate parameter, the q-value, was calculated using the q-value package provided by Bioconductor to correct for the multi-testing problem and was used to identify significantly differentially expressed genes [99]. A q-value cutoff of 0.05 was chosen to increase the likelihood of identifying informative changes in transcript levels but keep the risk of finding false positives low. Fold changes were obtained from the differences between the least square means obtained in the second ANOVA step of the analysis. Microarray data are compliant with the Minimum Information About a Microarray Experiment guidelines and the experimental data were deposited into the National Center for Biotechnology Information's Gene Expression Omnibus with super-series accession number GSE29269 and subseries numbers GSE29204 and GSE29268.

### Analyses of Functional Gene Categories using MapMan

Analysis of the microarray data using MapMan was essentially as previously described [100], [101]. For our analysis, we used q-values, adjusted for directional change (similar to [102]), rather than fold-changes. For each set of microarray data (MFA or AA), the q-values for the complete list of loci represented on the microarray were log-transformed. The sign of the direction of fold-change in gene transcript was assigned to the q-values to allow assessment of both magnitude of the q-value and whether transcript level increased or decreased [101]. The pathway diagrams and BINs used were those available in ImageAnnotator 2.0.0 (http://mapman.gabipd.org). The Wilcoxon-Mann-Whitney rank sum test using the Benjamini-Hochberg correction for false discovery, part of the ImageAnnotator analysis, was used to assign probabilities to BINs showing whether the average BIN response (q-value rank and direction) was significantly different from the average responses of all the other BINs in a data set [101]. We selected BINs having probabilities from the rank sum test of adjusted p<0.05 for further analyses. The overall direction of transcript level change, if any, was determined from counts of the number of genes with transcripts either increasing or decreasing.

### Cluster Analysis

Expression of 1316 genes was statistically significantly (q≤0.05) either up-regulated or down-regulated following AA treatment. By the same criteria, 606 genes were deemed MFA-responsive. These genes were used in a cluster analysis of transcript levels of the same genes during biotic and abiotic stress treatments. From public databases (Resource S5), 46 experiments were selected that used leaf or seedling treatment, and Affymetrix ATH1 or comparable arrays containing probes for loci covering nearly the entire genome. The treatment and control data were downloaded. The log_2_-transformed expression ratios were derived for all available replicates then averaged. From these data sets, the AA- or MFA-responsive genes that are represented in all array types were used in the respective cluster analyses. When AA-responsive genes were the query set, MFA treatment data were included in the analysis, and vice versa. The finalized data sets from these experiments were compared using Cluster [103]. Hierarchical Clustering/Average Linkage Clustering was used to generate array clusters based on Pearson correlation coefficients derived by Cluster. The clusters (Fig. 7) and heat maps (Resources S7 to S15) were visualized using Java TreeView (http://sourceforge.net/projects/jtreeview/). The Pearson correlation coefficients derived by Cluster between experiments are listed in Resource S6.

## Supporting Information

Resource S1 **Genes oppositely regulated during AA and MFA treatments.**
(XLS)

Resource S2 **MapMan BINs with rank sum adjusted probabilities of p<0.05 identified for the MFA- and AA-treatment q-value data sets.**
(XLS)

Resource S3 **Diagramatic representation of selected statistically significant functional gene categories (BINs) with cytochrome pathway inhibition by AA (left) or TCA cycle inhibition by MFA (right).** The BINs have been excerpted from MapMan diagrams. The logged q-value is shown for each gene (a small square) with the direction of transcript level change indicated by color, blue indicating an increase, and red indicating a decrease. All the BINs were standardized to the same range of the color scale. On the scale, values less than −1.3 or greater than 1.3 correspond to q-values of 0.05 or less.
(TIF)

Resource S4 **Fold-change and q-value data for genes in BINs that appear in** **Figs. 5** **and** **6** **and/or are discussed in the text.**
(XLS)

Resource S5 **List of experiments used for cluster analyses.**
(XLS)

Resource S6 **Pearson correlation coefficients for cluster analyses using genes with significantly altered expression (q≤0.05) by cytochrome pathway inhibition with AA or TCA cycle inhibition with MFA.**
(XLS)

Resource S7 **Heat map of gene expression data from all experiments used in the cluster analyses arranged by their associations as determined by the Cluster program using genes whose expression was altered in expression (q≤0.05) by AA.**
(TIF)

Resource S8 **Heat map of gene expression data from all experiments used in the cluster analyses arranged by their associations as determined by the Cluster program using genes whose expression was altered in expression (q≤0.05) by AA.**
(TIF)

Resource S9 **Heat map of gene expression data from all experiments used in the cluster analyses arranged by their associations as determined by the Cluster program using genes whose expression was altered in expression (q≤0.05) by AA.**
(TIF)

Resource S10 **Heat map of gene expression data from all experiments used in the cluster analyses arranged by their associations as determined by the Cluster program using genes whose expression was altered in expression (q≤0.05) by AA.**
(TIF)

Resource S11 **Heat map of gene expression data from all experiments used in the cluster analyses arranged by their associations as determined by the Cluster program using genes whose expression was altered in expression (q≤0.05) by AA.**
(TIF)

Resource S12 **Heat map of gene expression data from all experiments used in the cluster analyses arranged by their associations as determined by the Cluster program using genes whose expression was altered in expression (q≤0.05) by AA.**
(TIF)

Resource S13 **Heat maps of gene expression data from all experiments used in the cluster analyses arranged by their associations as determined by the Cluster program using genes whose expression was altered in expression (q≤0.05) by MFA.**
(TIF)

Resource S14 **Heat maps of gene expression data from all experiments used in the cluster analyses arranged by their associations as determined by the Cluster program using genes whose expression was altered in expression (q≤0.05) by MFA.**
(TIF)

Resource S15 **Heat maps of gene expression data from all experiments used in the cluster analyses arranged by their associations as determined by the Cluster program using genes whose expression was altered in expression (q≤0.05) by MFA.**
(TIF)
