# Supplementary figures and images for: Comparison of Intact Arabidopsis thaliana Leaf Transcript Profiles during Treatment with Inhibitors of Mitochondrial Electron Transport and TCA Cycle
Source: PLoS One. 2012 Sep 18;7(9):e44339. doi: 10.1371/journal.pone.0044339 (PMC3445595; doi:10.1371/journal.pone.0044339)

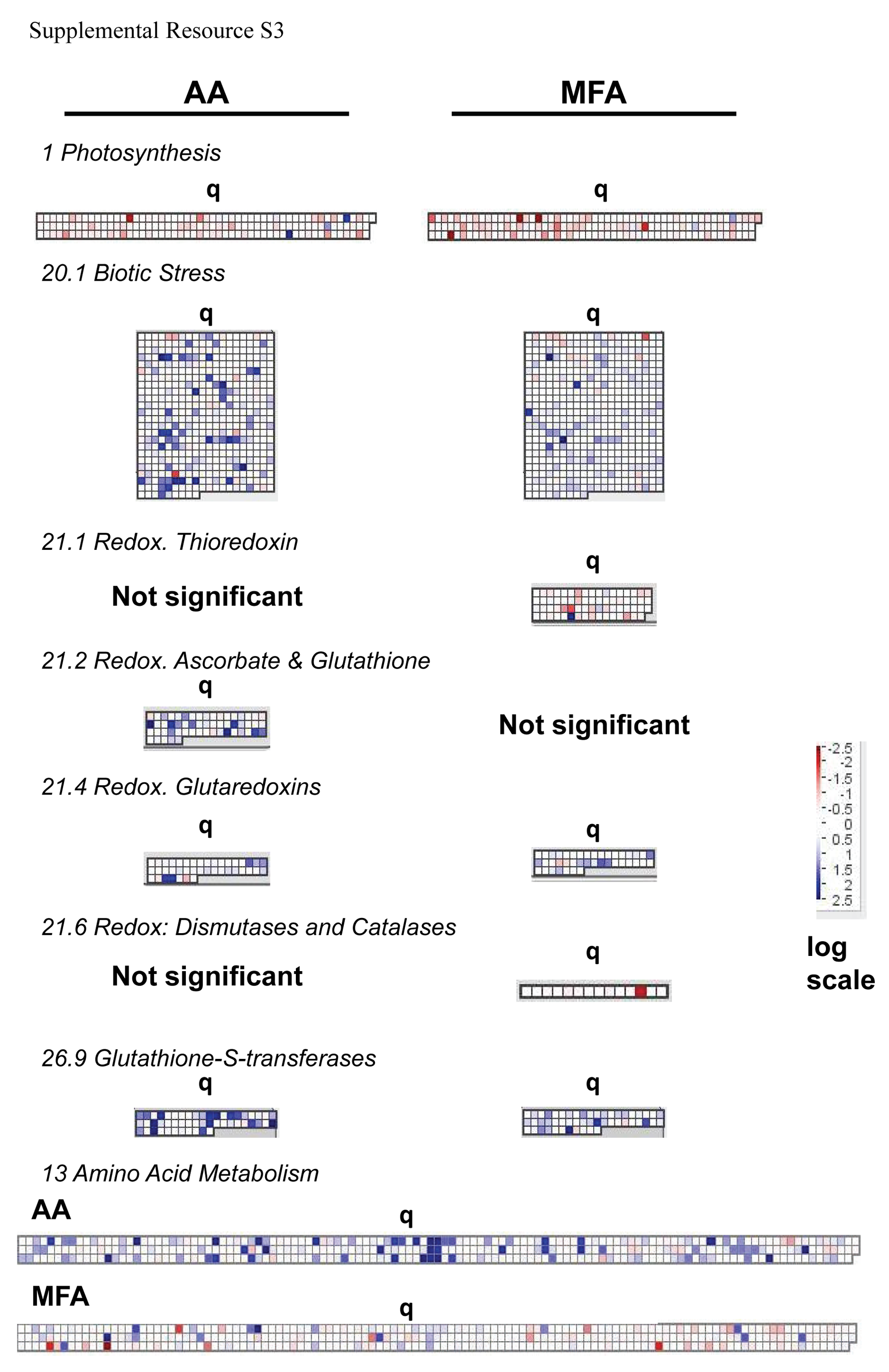

Supplement: Resource S3 — Diagramatic representation of selected statistically significant functional gene categories (BINs) with cytochrome pathway inhibition by AA (left) or TCA cycle inhibition by MFA (right). The BINs have been excerpted from MapMan diagrams. The logged q-value is shown for each gene (a small square) with the direction of transcript level change indicated by color, blue indicating an increase, and red indicating a decrease. All the BINs were standardized to the same range of the color scale. On the scale, values less than −1.3 or greater than 1.3 correspond to q-values of 0.05 or less. (TIF) [file pone.0044339.s003.tif]

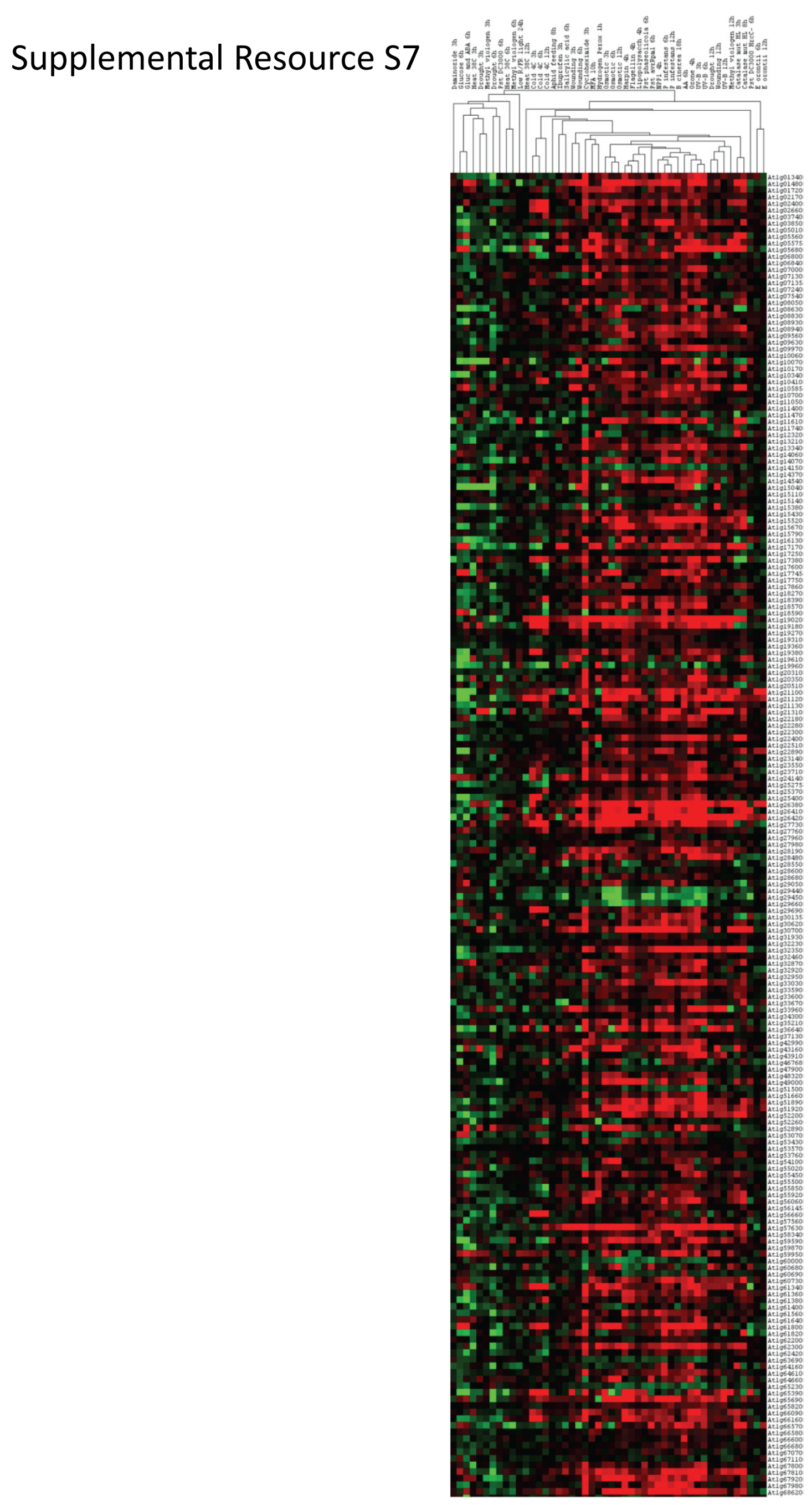

Supplement: Resource S7 — Heat map of gene expression data from all experiments used in the cluster analyses arranged by their associations as determined by the Cluster program using genes whose expression was altered in expression (q≤0.05) by AA. (TIF) [file pone.0044339.s007.tif]

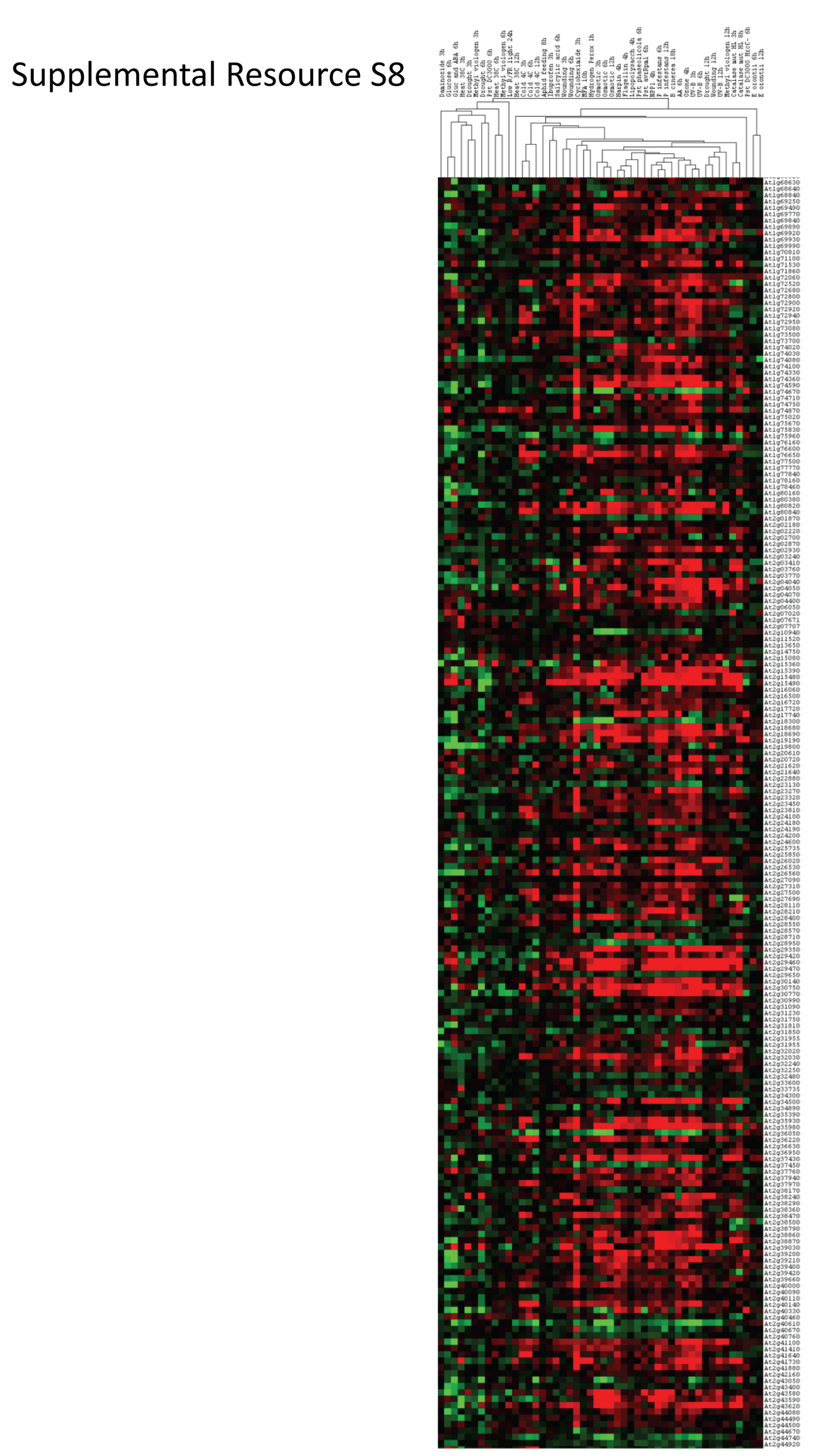

Supplement: Resource S8 — Heat map of gene expression data from all experiments used in the cluster analyses arranged by their associations as determined by the Cluster program using genes whose expression was altered in expression (q≤0.05) by AA. (TIF) [file pone.0044339.s008.tif]

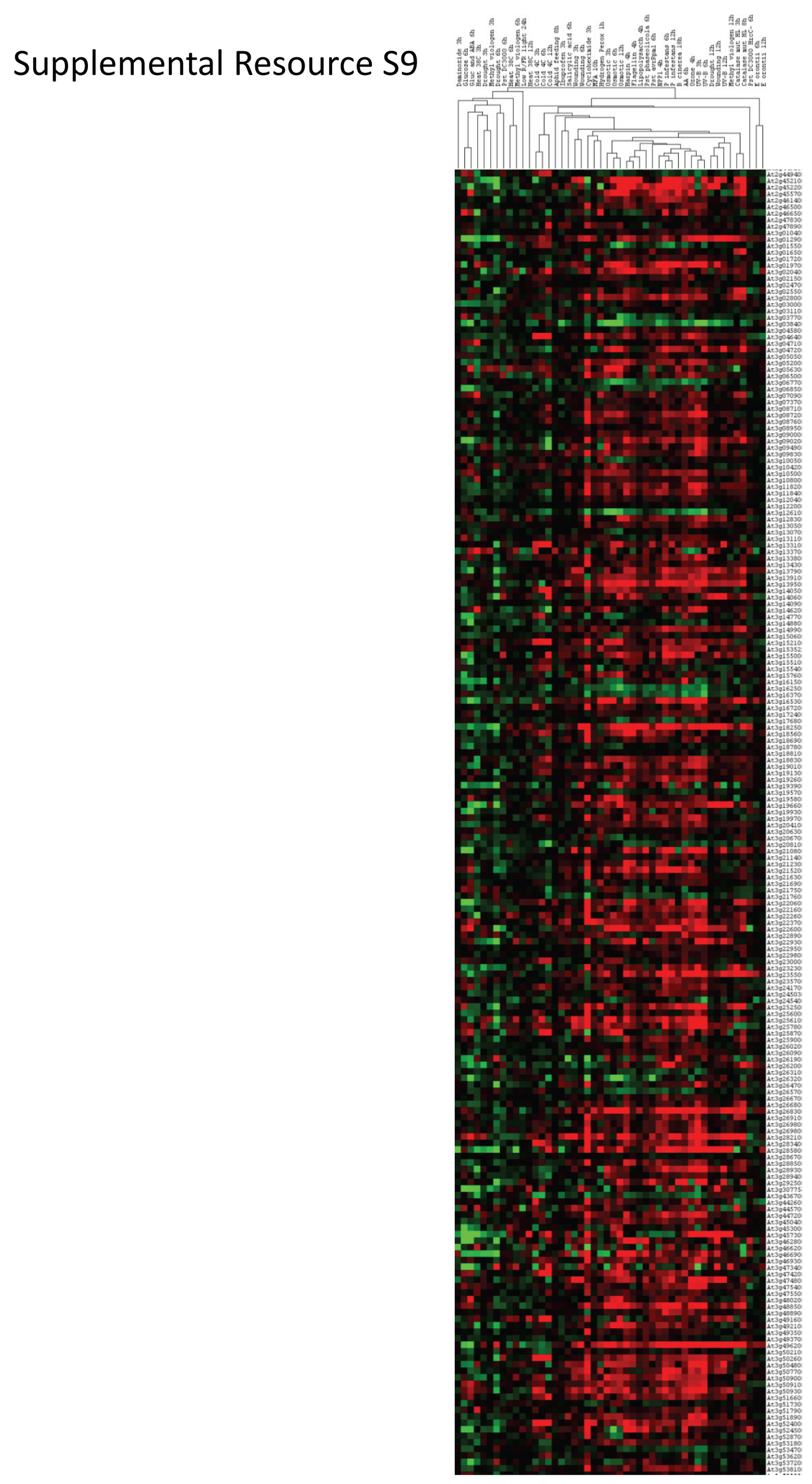

Supplement: Resource S9 — Heat map of gene expression data from all experiments used in the cluster analyses arranged by their associations as determined by the Cluster program using genes whose expression was altered in expression (q≤0.05) by AA. (TIF) [file pone.0044339.s009.tif]

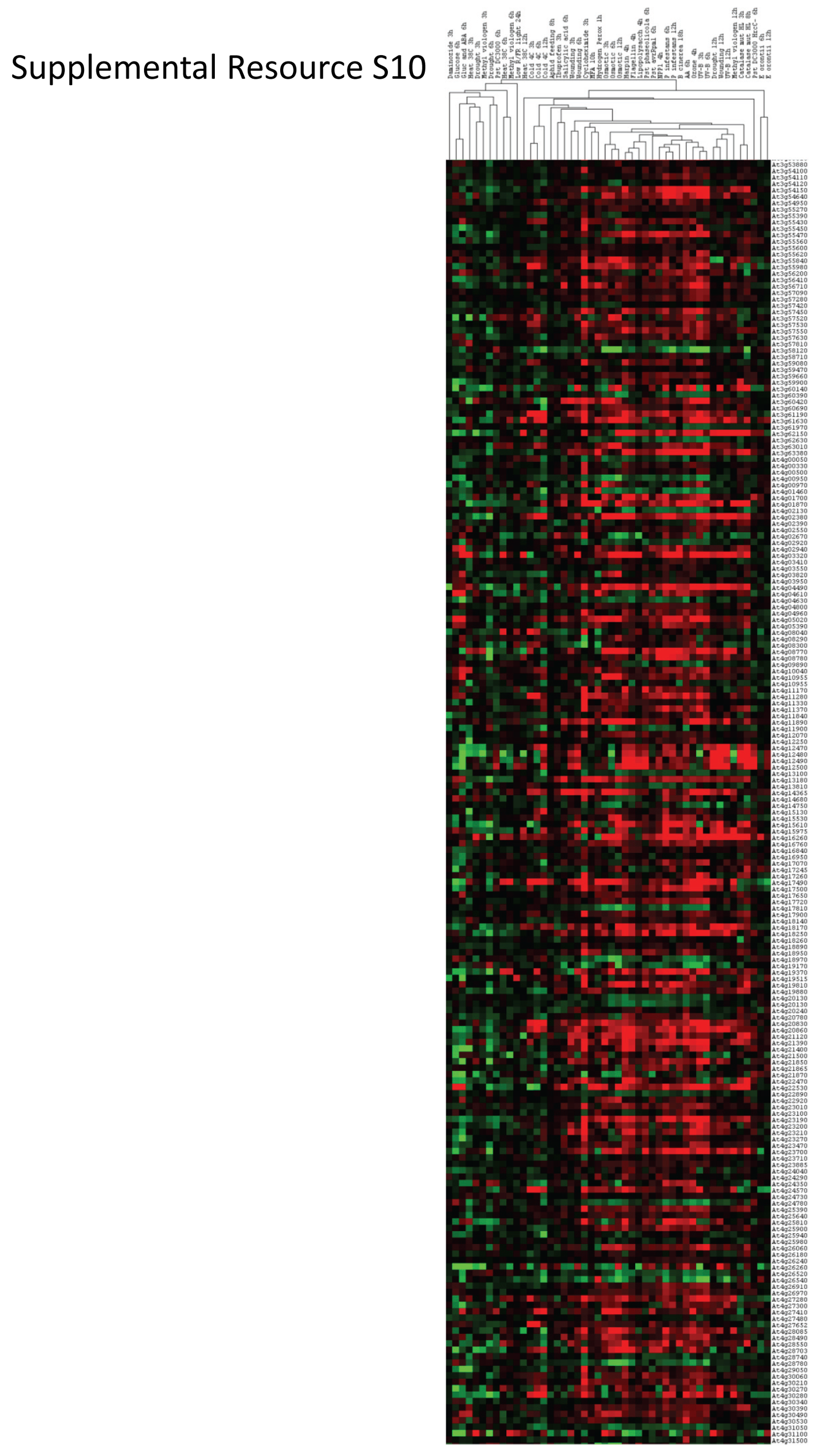

Supplement: Resource S10 — Heat map of gene expression data from all experiments used in the cluster analyses arranged by their associations as determined by the Cluster program using genes whose expression was altered in expression (q≤0.05) by AA. (TIF) [file pone.0044339.s010.tif]

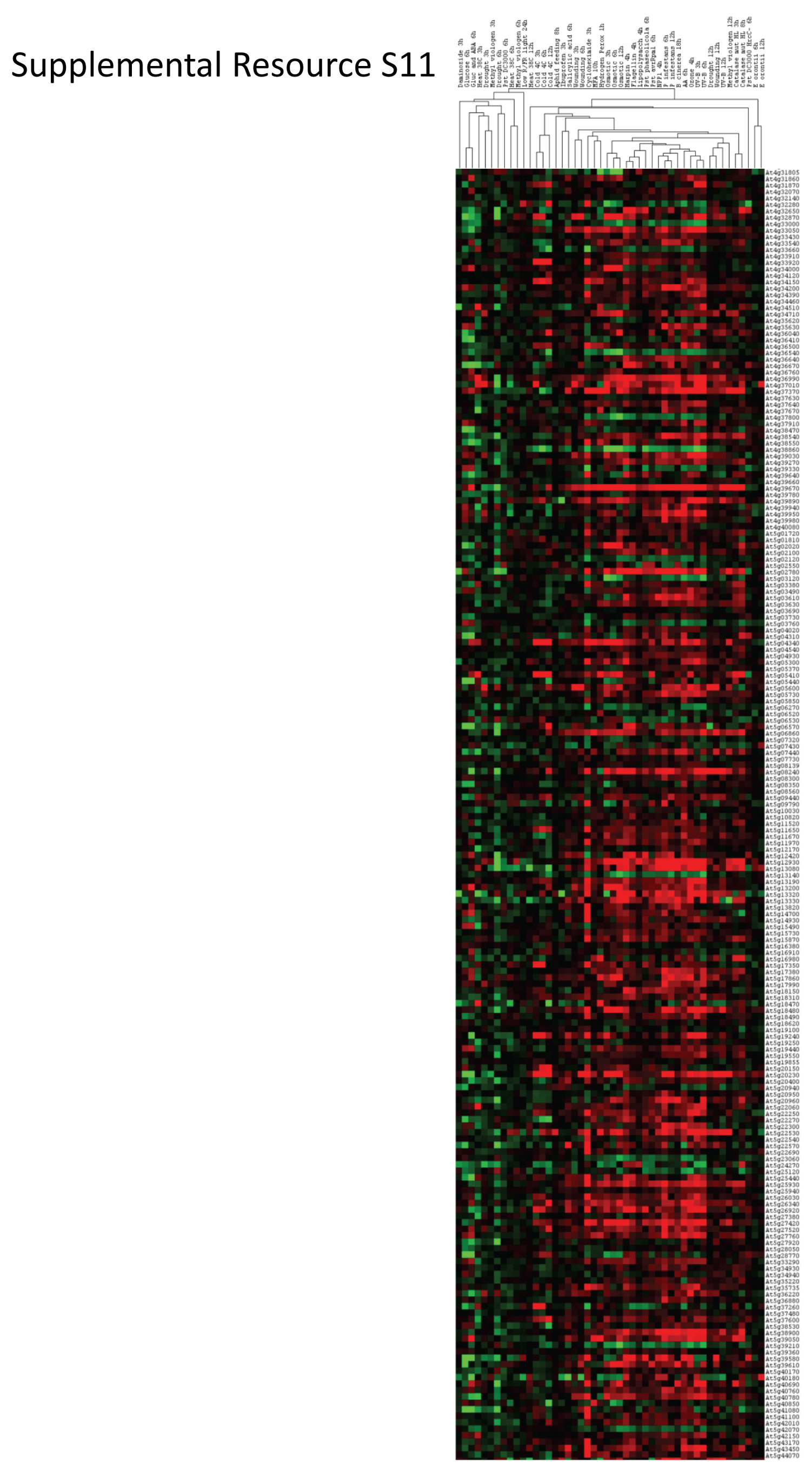

Supplement: Resource S11 — Heat map of gene expression data from all experiments used in the cluster analyses arranged by their associations as determined by the Cluster program using genes whose expression was altered in expression (q≤0.05) by AA. (TIF) [file pone.0044339.s011.tif]

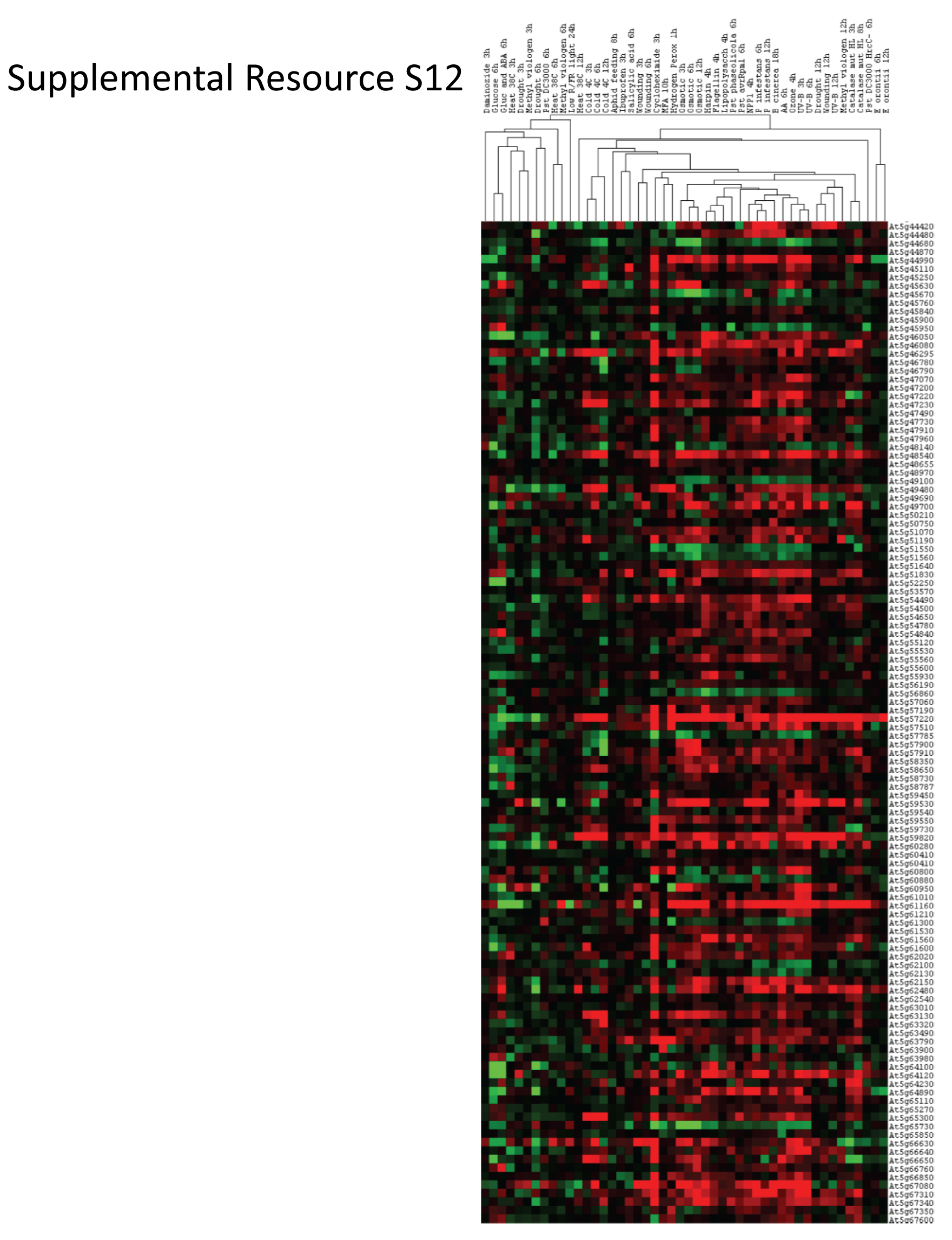

Supplement: Resource S12 — Heat map of gene expression data from all experiments used in the cluster analyses arranged by their associations as determined by the Cluster program using genes whose expression was altered in expression (q≤0.05) by AA. (TIF) [file pone.0044339.s012.tif]

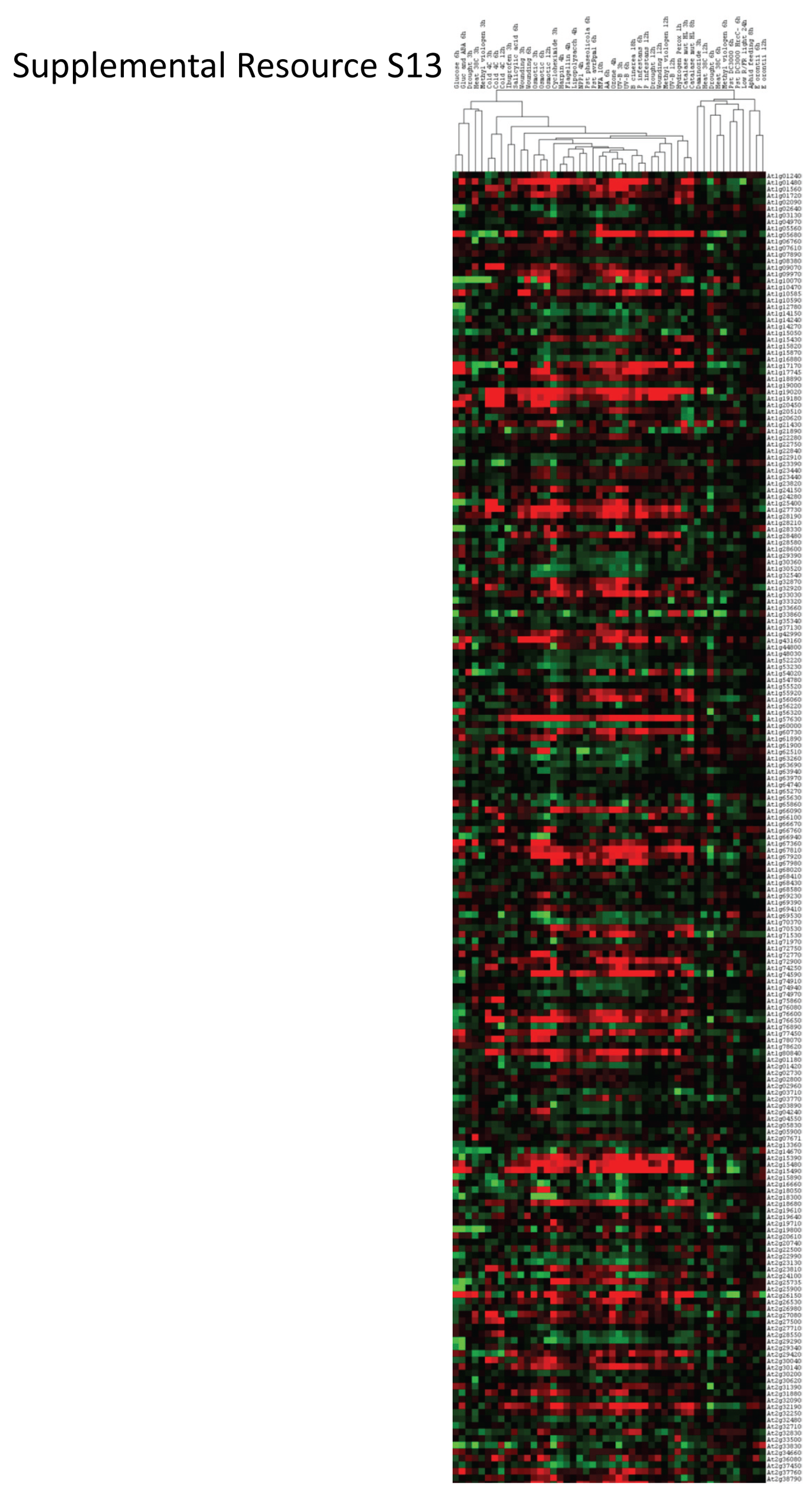

Supplement: Resource S13 — Heat maps of gene expression data from all experiments used in the cluster analyses arranged by their associations as determined by the Cluster program using genes whose expression was altered in expression (q≤0.05) by MFA. (TIF) [file pone.0044339.s013.tif]

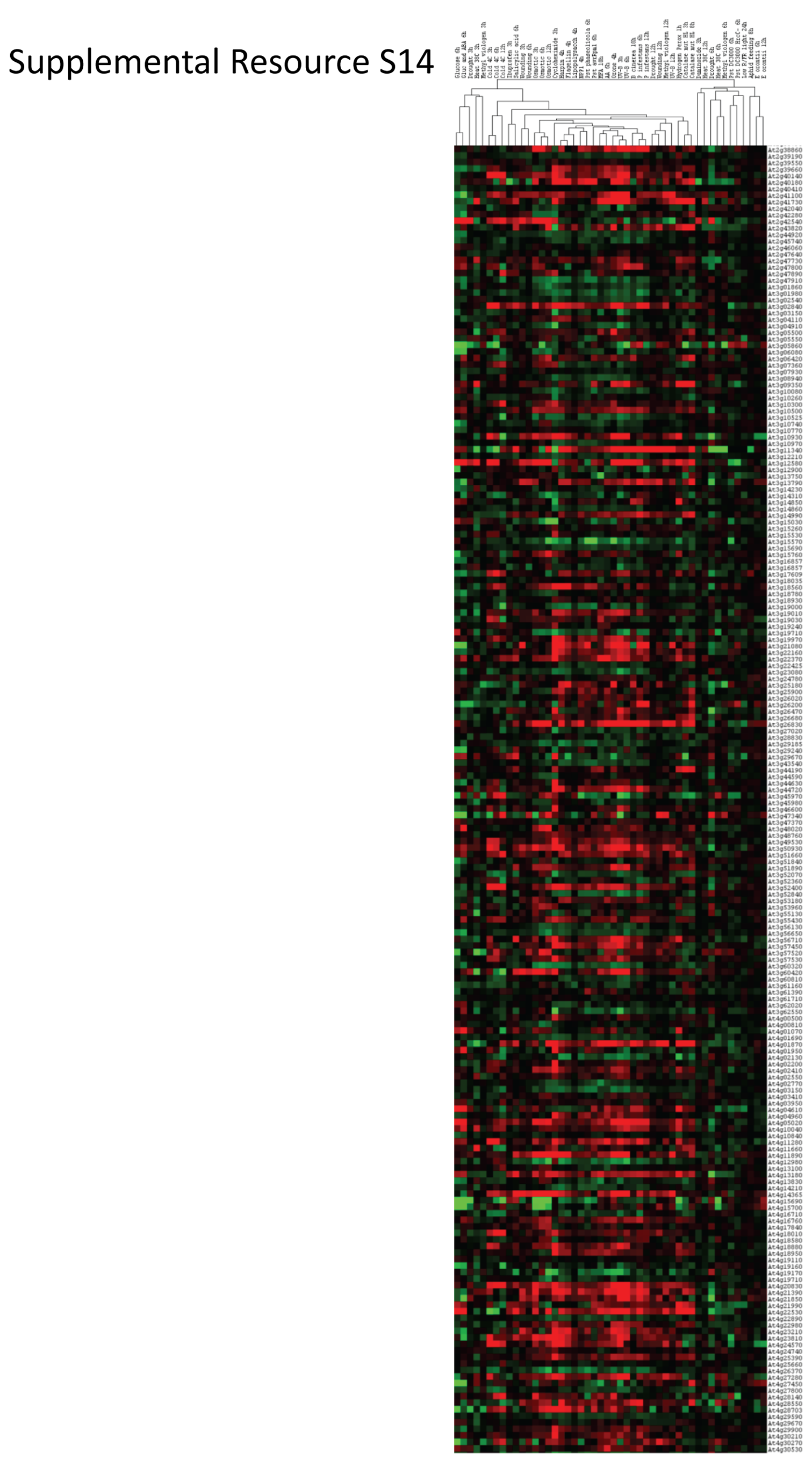

Supplement: Resource S14 — Heat maps of gene expression data from all experiments used in the cluster analyses arranged by their associations as determined by the Cluster program using genes whose expression was altered in expression (q≤0.05) by MFA. (TIF) [file pone.0044339.s014.tif]

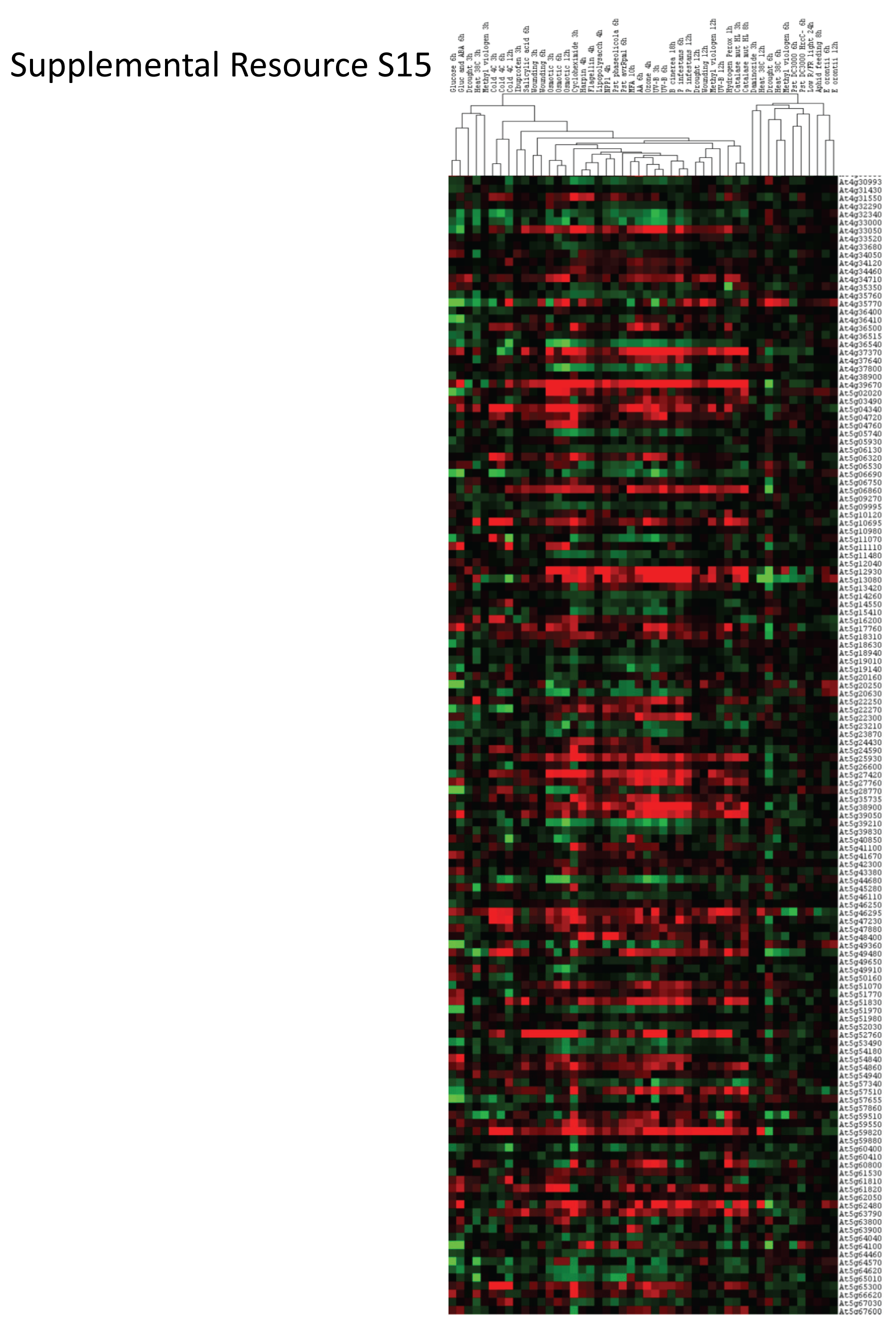

Supplement: Resource S15 — Heat maps of gene expression data from all experiments used in the cluster analyses arranged by their associations as determined by the Cluster program using genes whose expression was altered in expression (q≤0.05) by MFA. (TIF) [file pone.0044339.s015.tif]
